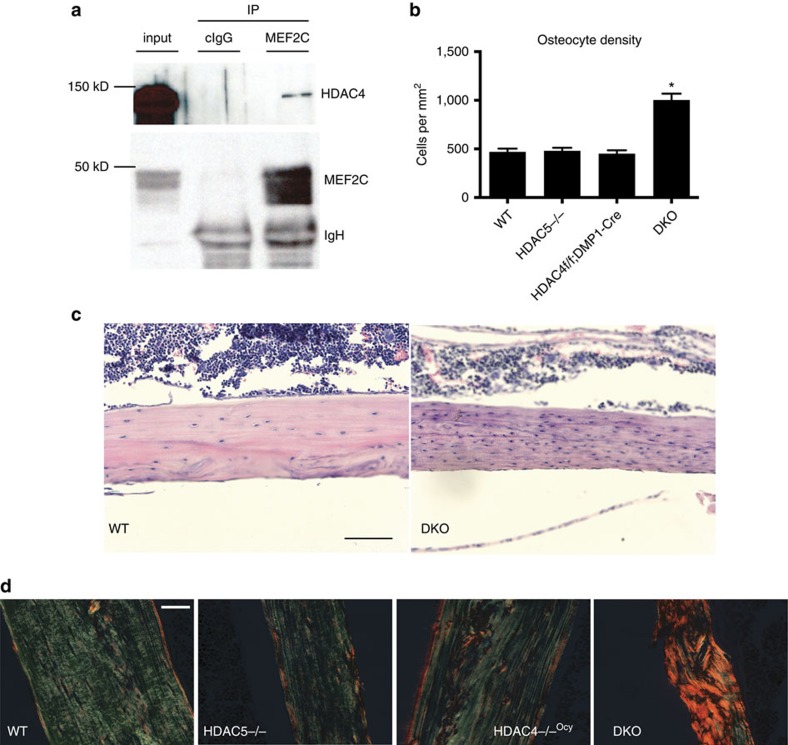# Corrigendum: SIKs control osteocyte responses to parathyroid hormone

**DOI:** 10.1038/ncomms14745

**Published:** 2017-02-22

**Authors:** Marc N. Wein, Yanke Liang, Olga Goransson, Thomas B. Sundberg, Jinhua Wang, Elizabeth A. Williams, Maureen J. O'Meara, Nicolas Govea, Belinda Beqo, Shigeki Nishimori, Kenichi Nagano, Daniel J. Brooks, Janaina S. Martins, Braden Corbin, Anthony Anselmo, Ruslan Sadreyev, Joy Y. Wu, Kei Sakamoto, Marc Foretz, Ramnik J. Xavier, Roland Baron, Mary L. Bouxsein, Thomas J. Gardella, Paola Divieti-Pajevic, Nathanael S. Gray, Henry M. Kronenberg

Nature Communications 7: Article number: 13176; DOI: 10.1038/ncomms13176 (2017); Published 10 19 2016; Updated 02 22 2017

In this article, there are errors in the labelling of the *y* axis in Fig. 1b. The labels '50', '100' 'and ‘150' should have been ‘500', '1,000' and ‘1,500', respectively. The correct version of this figure appears as Fig. 1 below.

## Figures and Tables

**Figure 1:**