# Supplementary material for: SIKs control osteocyte responses to parathyroid hormone
Source: Nat Commun. 2016 Oct 19;7:13176. doi: 10.1038/ncomms13176 (PMC5075806; doi:10.1038/ncomms13176)
Supplement: Supplementary Information — Supplementary Figures 1-9, Supplementary Tables 1-4, Supplementary Methods and Supplementary References [file ncomms13176-s1.pdf]

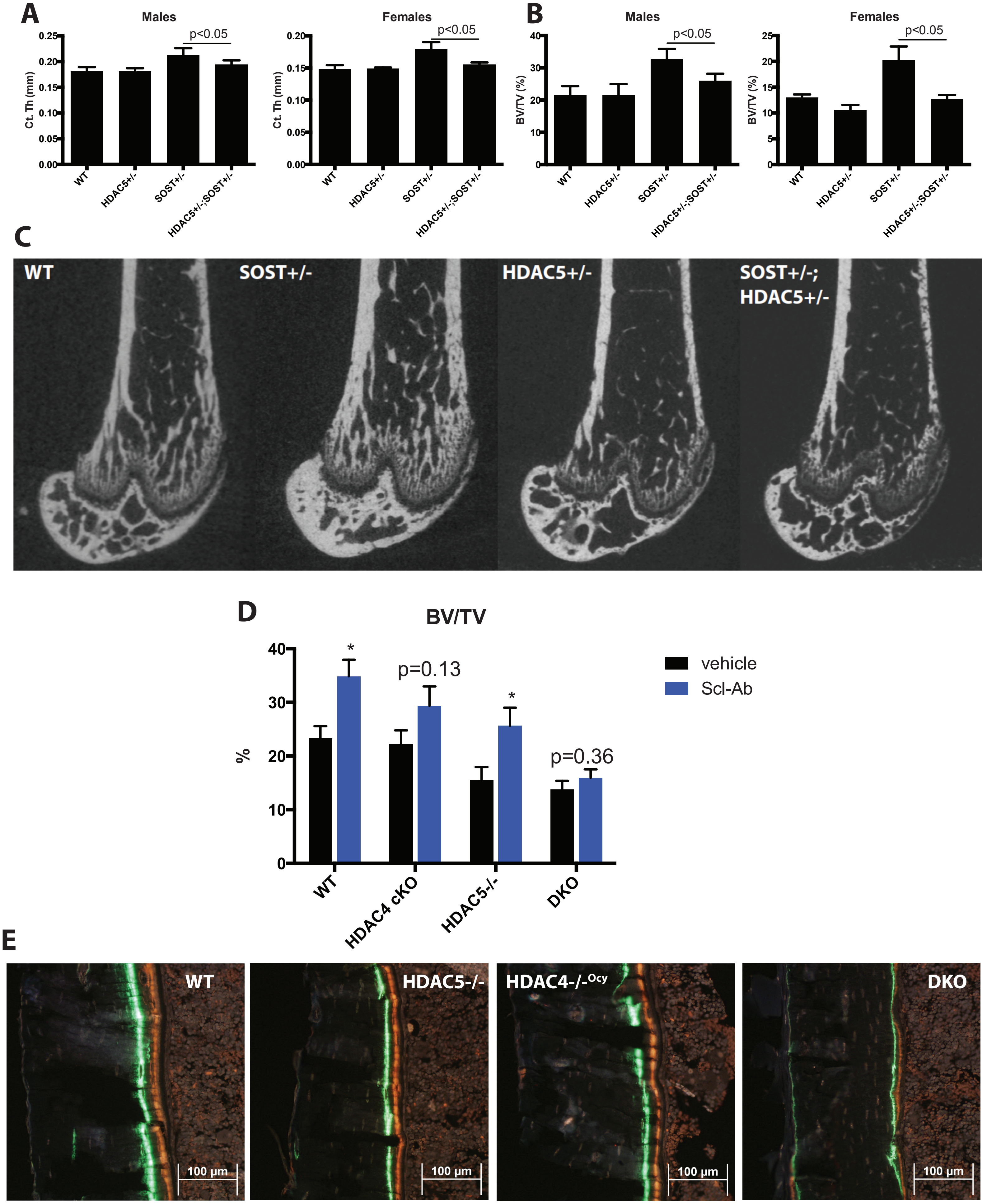

Supplementary Figure 1

Supplementary Figure 1. (A) Cortical and (B) trabecular micro-CT results from 8 week old mice of the indicated compound heterozygous genotype. The high bone mass observed in SOST+/- mice is not observed in SOST/HDAC5 compound heterozygotes. (C) Representative sagittal images from mice analyzed in (A) and (B). (D) Male WT (n=5), HDAC4^{OcyKO} (n=6), HDAC5-/- (n=5), and DKO (n=6) mice were treated with anti-sclerostin antibody (50 mg kg^{-1}) twice weekly from 2 to 8 weeks of age. Distal femur BV/TV was determined by micro-CT. 2 way ANOVA analysis revealed a significant interaction between genotype and Scl-Ab treatment, therefore post-hoc t tests were performed to determine effects of Scl Ab treatment within each genotype. Individual p values for each comparison are noted on the graph. (E) Dual calcein/demeclocycline images showing reduced endocortical bone formation in DKO mice.

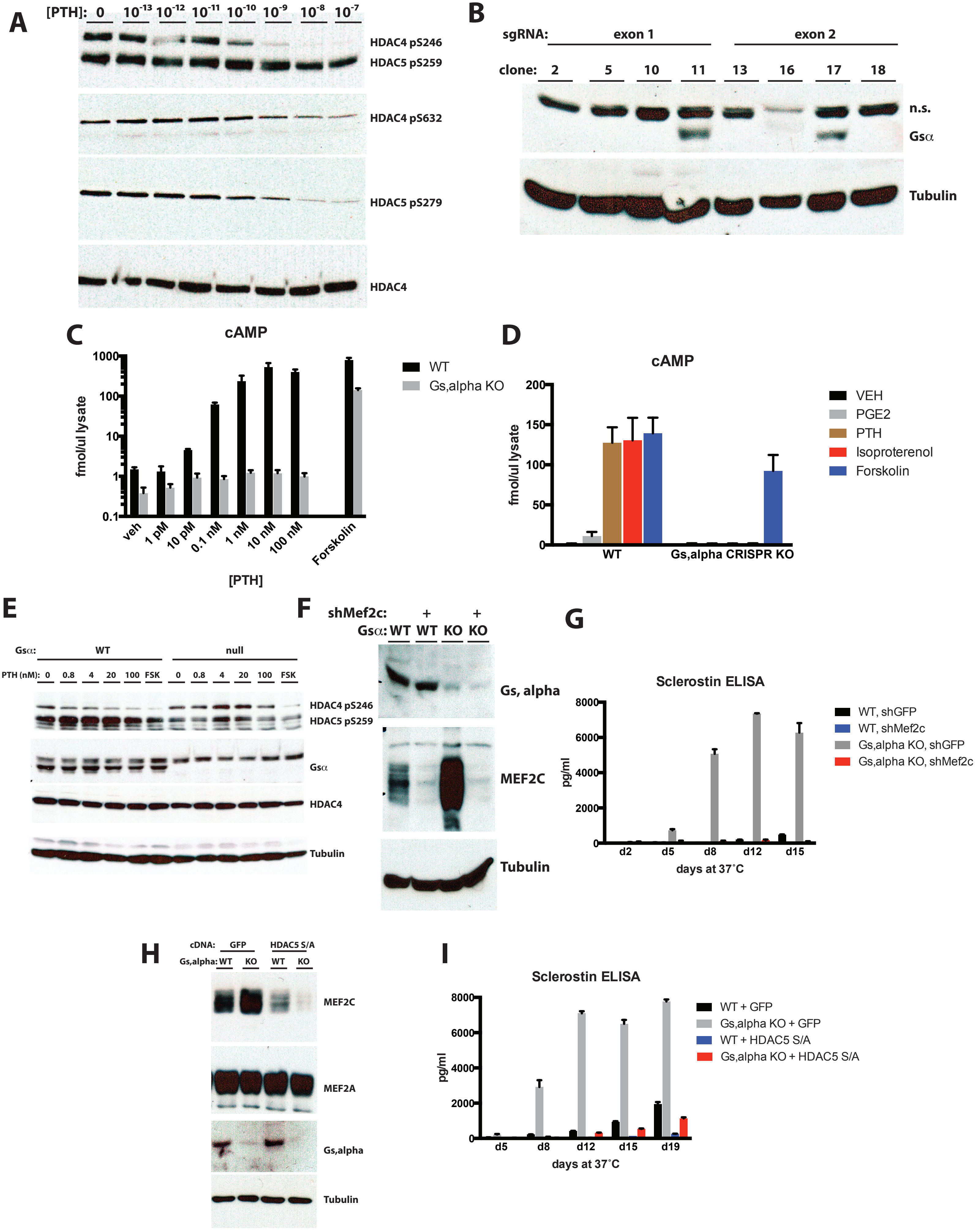

Supplementary Figure 2

Supplementary Figure 2. (A) Ocy454 cells were treated with the indicated concentrations of PTH for 30 minutes, followed by immunoblotting as indicated. (B) Immunoblots from individual single cell clones isolated after exposure to Gsα sgRNA/Cas9 targeting the indicated GNAS exon. Clones 11 and 17 show intact Gsα expression, while the other clones show no detectable Gsα protein. Clone 8, not shown here, also was isolated after exposure to the sgRNA sequence targeting GNAS exon 1. (C) WT and Gsα KO cells were treated with the indicated concentrations of PTH and cAMP levels were measured by RIA 20 minutes later. No detectable PTH-induced increases in cAMP were observed in cells lacking Gsα. (D) As in (C), except cells were treated with other agents known to stimulate cAMP production. (E) WT and Gsα null cells were treated with the indicated concentrations of PTH and analyzed by immunoblotting as in (A). (F) Cells lacking Gsα were infected with control or MEF2C shRNA lentiviruses, followed by immunoblotting as indicated. (G) Cells from (F) were allowed to differentiate for the indicated times at 37˚C, and sclerostin ELISAs were then performed from the conditioned medium. While Gsα KO cells showed increased sclerostin secretion, MEF2C shRNA abrogates this effect. (H) Cells lacking Gsα were infected with lentiviruses to over-express HDAC5 S/A (S259/498A) and analyzed by immunoblotting. (I) Cells from (H) were analyzed as in (G). HDAC5 S/A overexpression dramatically reduces sclerostin secretion by Gsα deficient cells.

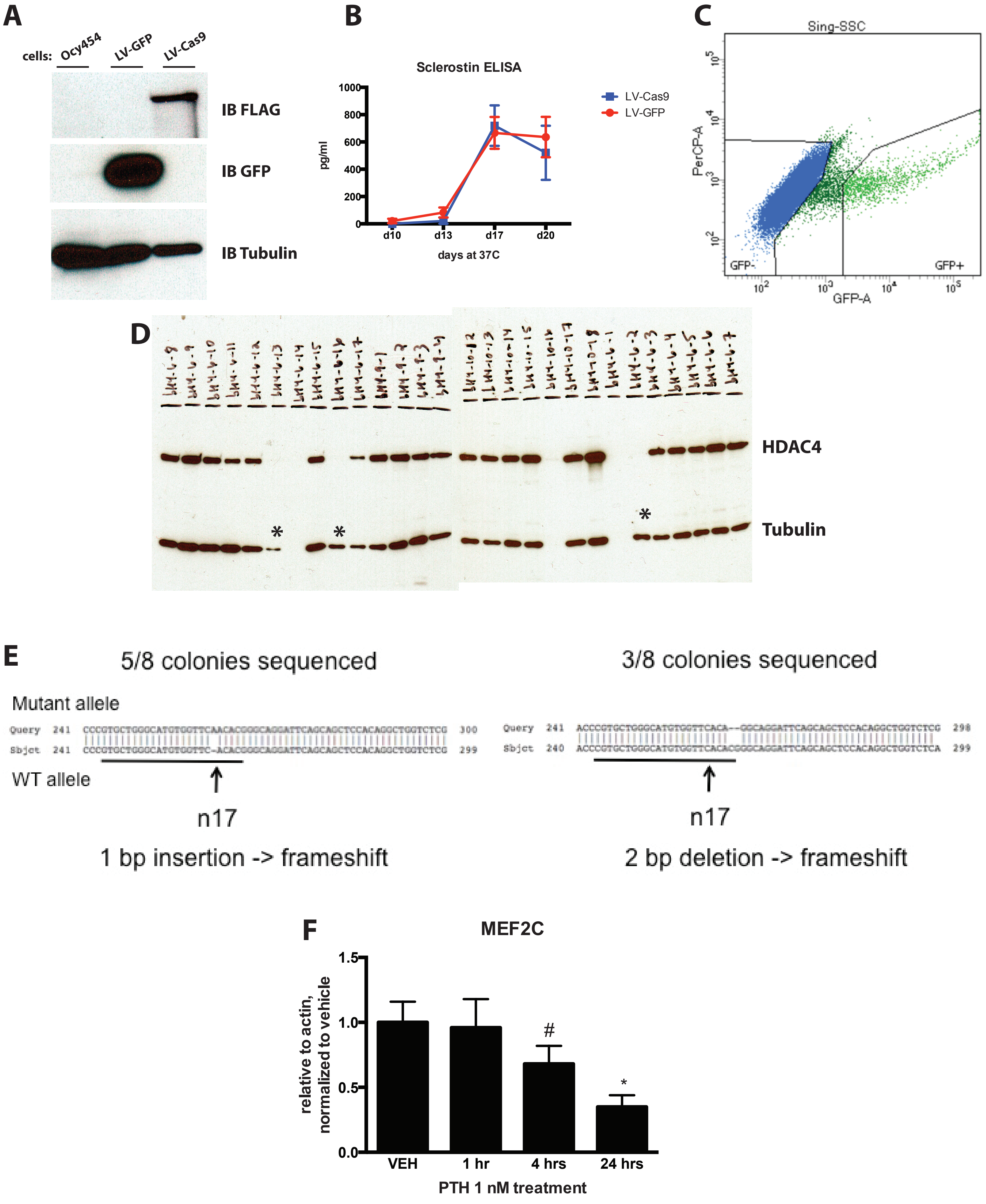

Supplementary Figure 3

Supplementary Figure 3. (A) Immunoblot showing Ocy454 cells uninfected with lentivirus, infected with control lentivirus (LV-GFP), or infected with lentivirus overexpressing FLAG-tagged Cas9. (B) Sclerostin ELISA demonstrating no effect of Cas9 expression on Ocy454 cell sclerostin secretion in the absence of sgRNA co-expression. (C) Ocy454 cells were transfected with PX458 plasmid which co-expresses a sgRNA of interest, Cas9, and eGFP. 48 hours later, eGFP positive cells were sorted by flow cytometry into 96 well plates at a density of 1 cell per well. Clones were identified, expanded, and analyzed by immunoblotting. (D) Representative immunoblot of single cell clones isolated after exposure to an sgRNA targeting HDAC4. Starred clones show no detectable HDAC4 protein. (E) Genomic DNA was isolated from individual HDAC4 deficient clones followed by allele-specific sequencing. As shown in the example here, cells without HDAC4 protein show bi-allelic HDAC4 insertion/deletions resulting in frameshift mutations. N17 refers to the 17th nucleotide within the 20mer sgRNA sequence, where insertions/deletions are most likely to occur. (F) Ocy454 cells were treated with PTH (1 nM) for the indicated times, and MEF2C transcript abundance was measured by RT-qPCR. # indicates $p<0.05$, and * indicates $p<0.01$ vs vehicle.

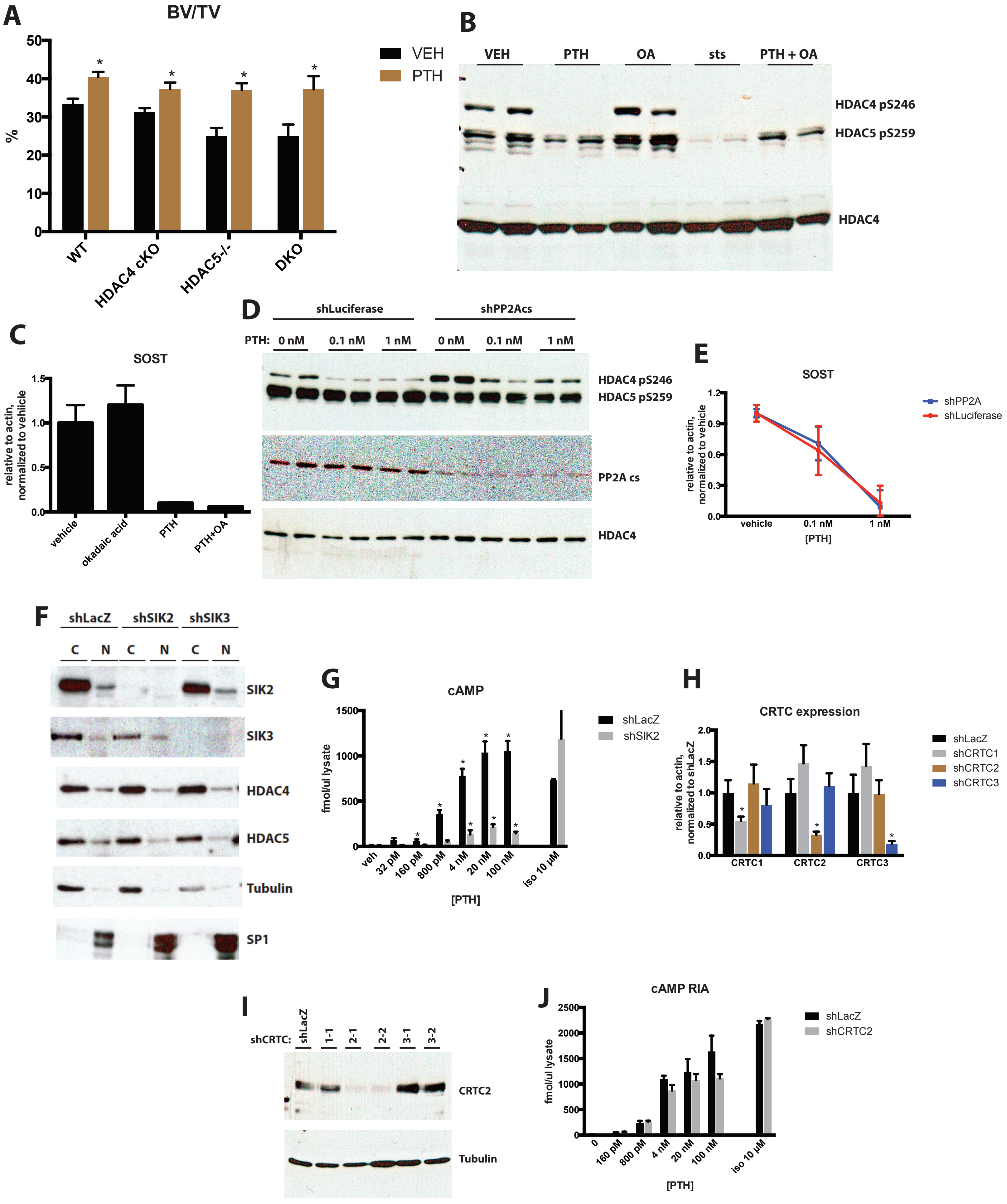

Supplementary Figure 4

Supplementary Figure 4. (A) 8 week old female mice of the indicated genotypes were treated with vehicle or hPTH (1-34, 100 mcg kg^{-1}) once daily, 5 days per week, for 4 weeks. Micro-CT analysis of bone volume fraction of the primary spongiosa is shown. 2 way ANOVA revealed a significant ($p<0.01$) interaction between genotype and drug treatment. Therefore, posthoc t tests were performed comparing effects of vehicle and PTH within each genotype. * indicates $p<0.01$. (B) Ocy454 cells were treated with vehicle, PTH (50 nM), okadaic acid (300 nM), staurosporine (sts, 1 µM), or PTH plus okadaic acid. When okadaic acid was used, cells were pre-treated with this agent for 20 minutes. 30 minutes later, whole cell lysates were obtained followed by immunoblotting as indicated. Okadaic acid does not block the ability of PTH to induce HDAC4/5 dephosphorylation. (C) Ocy454 cells were treated with okadaic acid (300 nM), PTH (1 nM), and both. 4 hours later, RNA was isolated and SOST transcript abundance was analyzed by RT-qPCR. (D) Ocy454 cells were infected with control (shLacZ) or PP2A catalytic subunit (c.s.) shRNA-expressing lentiviruses. Cells were then treated with the indicated concentrations of PTH for 30 minutes followed by immunoblotting as indicated. (E) Cells from (D) were treated with the indicated concentrations of PTH and SOST transcript abundance was measured by RT-qPCR 4 hours later. (F) Control, shSIK2, and shSIK3 Ocy454 cells were subjected to subcellular fractionation followed by immunoblotting for the indicated proteins. (G) Control (shLacZ) and shSIK2 cells were treated with the indicated concentrations of PTH or isoproterenol, and cAMP levels were determined by RIA. While shSIK2 cells show reduced cAMP levels at all doses compared to control cells, significant upregulation (versus vehicle) in these cells is noted at doses above 4 nM. (H) Ocy454 cells were infected with shRNAs targeting CRTC1, CRTC2, and CRTC3. Knockdown efficiency and specificity for each gene was then measured by RT-qPCR. (I) CRTC2 shRNAs effectively reduce CRTC2 protein levels. (J) CRTC2 knockdown cells show normal PTH-induced cAMP generation as measured by radioimmunoassay.

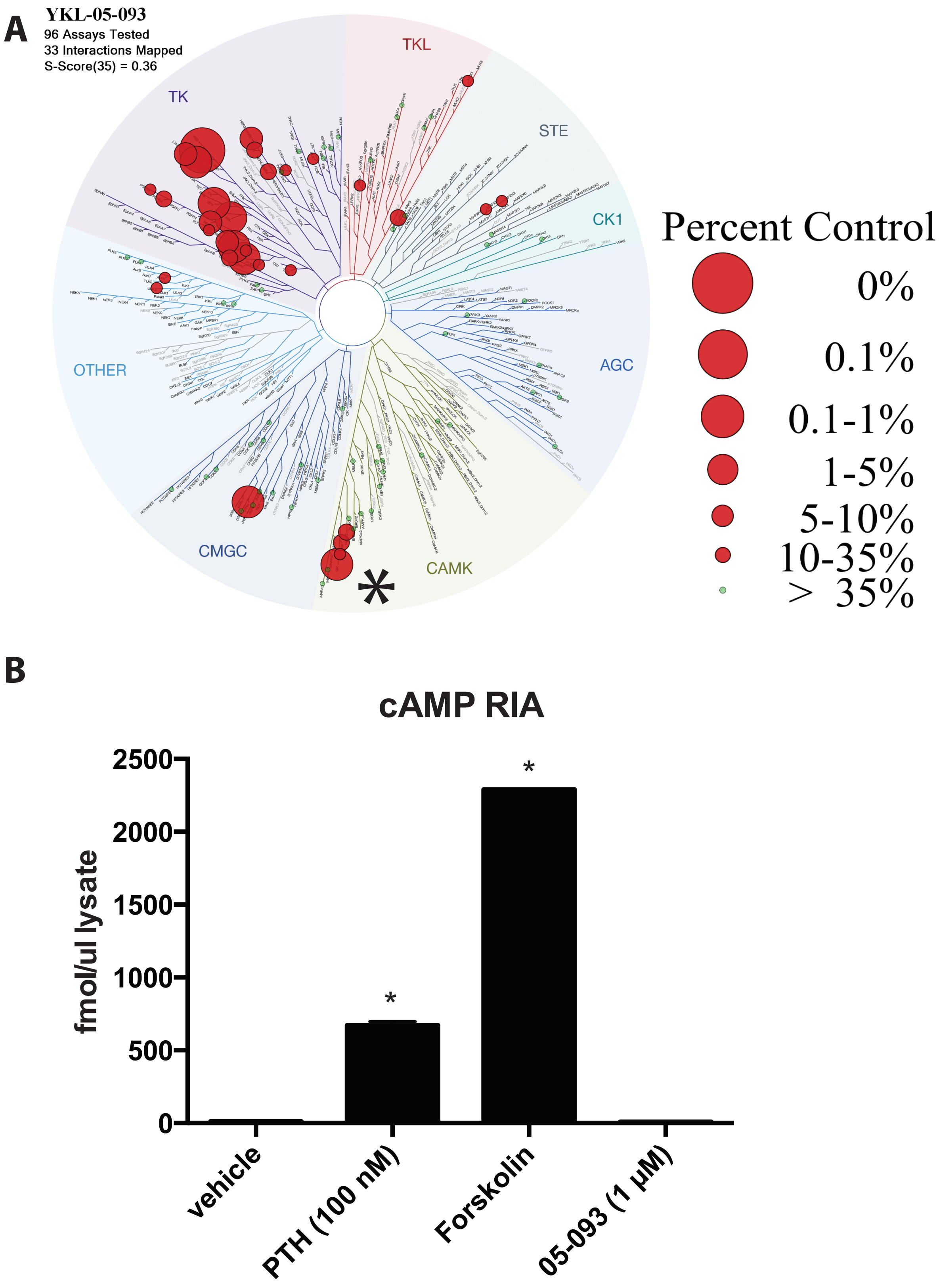

Supplementary Figure 5

Supplementary Figure 5. (A) Dendrogram showing effects of YKL-05-093 on different classes of kinases. The location of SIK kinases is denoted with an asterix. SIK refers to SIK1, and QSK refers to SIK3 in these assays. See Supplemental Table 3 for more details. Kinase group names follow standard nomenclature: AGC (containing PKA, PKG, PKC families), CAMK (calcium/calmodulin-dependent protein kinases), CK1 (casein kinase 1), CMGC (containing CDK, MAPK, GSK3, CLK families), STE (homologs of yeast sterile 7, sterile 11, and sterile 20 kinases), TK (tyrosine kinase), and TKL (tyrosine kinase-like). Image generated using TREE*spot*™ Software Tool and reprinted with permission from KINOME*scan*®, a division of DiscoveRx Corporation, © DISCOVERX CORPORATION 2010. (B) Ocy454 cells were treated with PTH, forskolin, or YKL-05-093, and cAMP levels were measured by RIA 20 minutes later. YKL-05-093 does not induce cAMP generation.

A

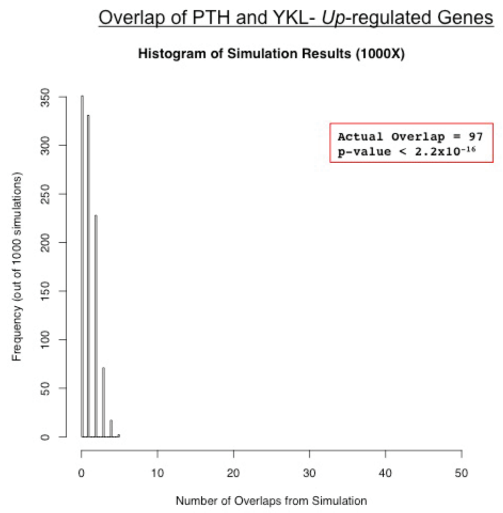

B

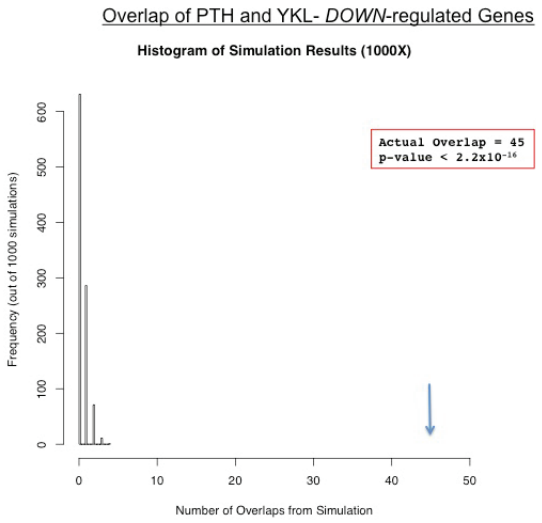

C

GO Term	FDR value (coverage)
Cell chemotaxis	2.04e-5 (10/174)
Blood vessel morphogenesis	2.04e-5 (12/278)
Ossification	8.99e-5 (11/275)
Muscle organ development	1.54e-4 (11/298)
Relaxation of cardiac muscle	2.1e-4 (4/10)

D

GO Term	FDR value (coverage)
Skeletal muscle tissue development	6.052e-6 (9/166)
Blood vessel morphogenesis	1.69e-5 (10/278)
Muscle organ development	2.2e-5 (10/298)
Skeletal muscle cell differentiation	2.35e-3 (5/61)
Regulation of muscle tissue development	2.78e-2 (5/113)

E

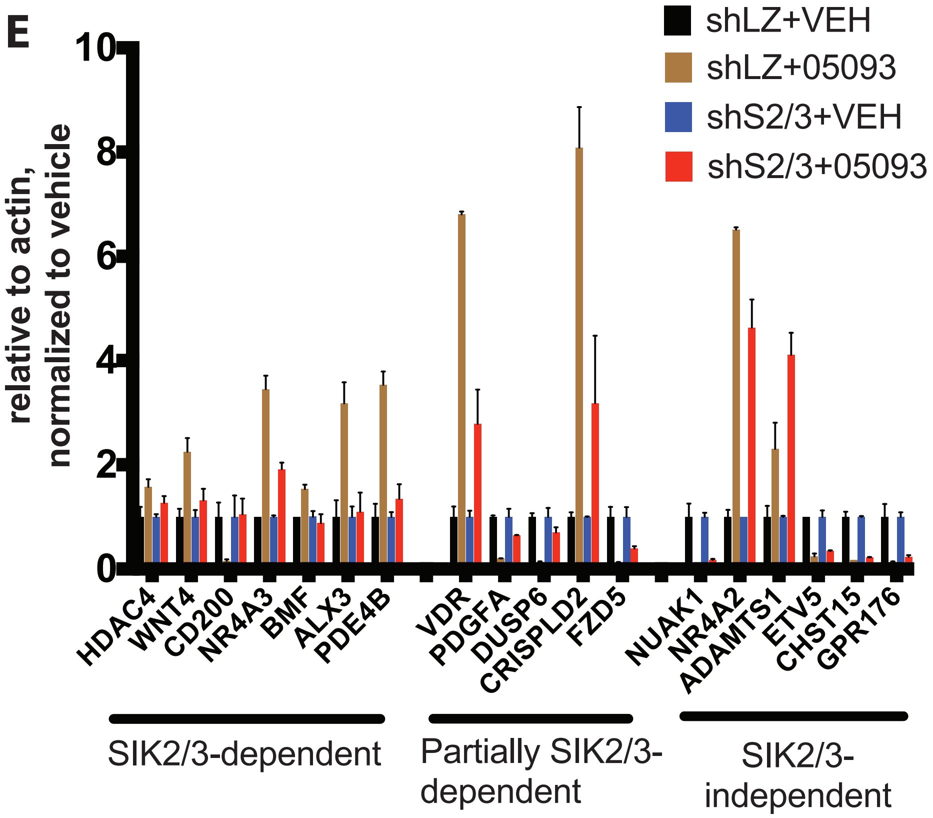

Supplementary Figure 6

Supplementary Figure 6. (A, B) Simulation results demonstrating that the overlap between the group of genes co-regulated in the same direction by PTH and YKL-05-093 is not due to random chance. (C, D) Gene ontology analysis of genes up- or down-regulated by both PTH and YKL-05-093. (E) Control and shSIK2/3 cells were treated with vehicle or YKL-05-093 (0.5 μM) for 4 hours, and the indicated genes were measured by RT-qPCR. Genes are categorized based on the dependence of SIK2/3 for the ability of YKL-05-093 to regulate their expression. Genes that are SIK2/3-dependent show no YKL-05-093-induced regulation in SIK2/3-deficient cells. Genes that are partially SIK2/3-dependent show blunted regulation by YKL-05-093 in SIK2/3-deficient cells. Genes that are SIK2/3-independent show normal YKL-05-093-induced regulation in SIK2/3-deficient cells. Therefore, the regulation of these genes is likely due to cellular targets of YKL-05-093 other than SIK2/3.

Half life in 1 mg/ml hepatic microsomes

Compound ID	Mouse
**YKL-04-114**	13.8
**YKL-05-093**	22.7
**HG-9-91-01**	13.0

Supplementary Figure 7

Supplementary Figure 7. The half-life of the indicated compound was measured in murine hepatic microsomes. Note the improved half-life of YKL-05-093 compared to YKL-04-114 and HG-9-91-01.

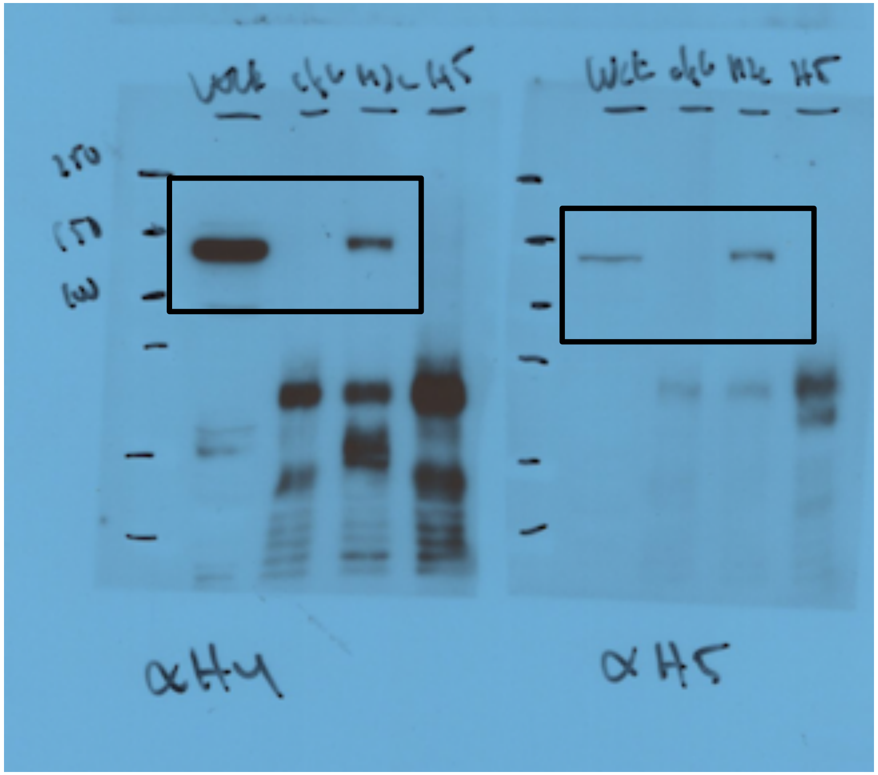

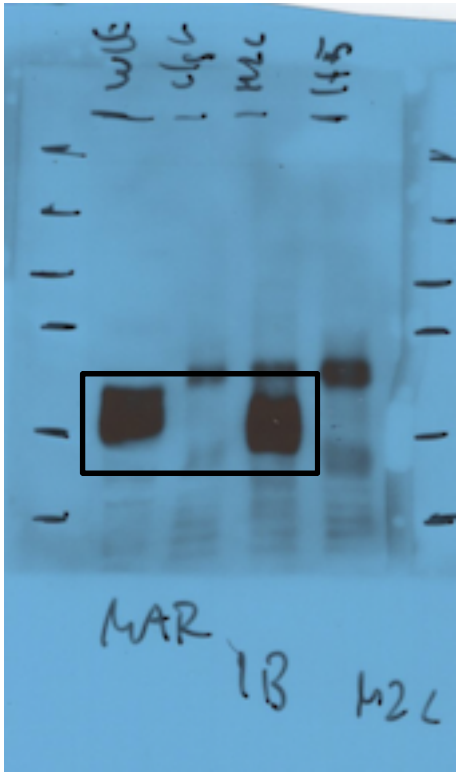

Figure 1A

Supplementary Figure 8

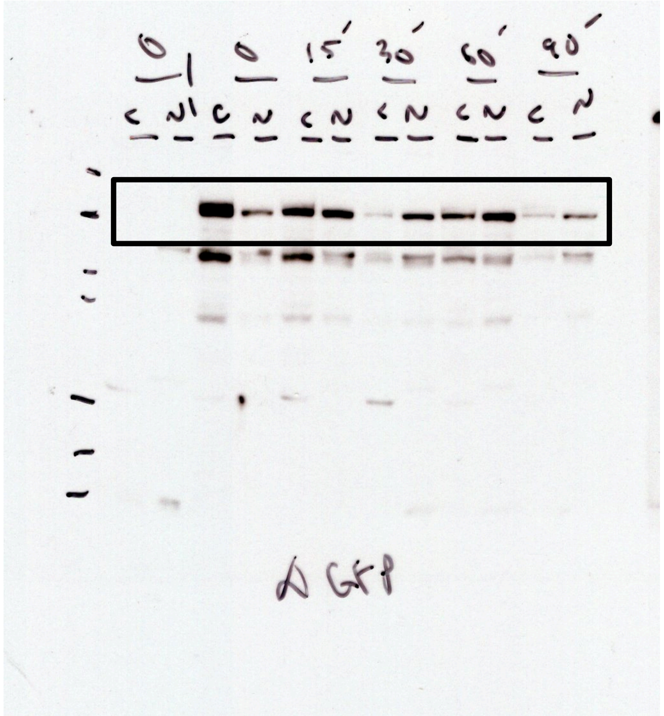

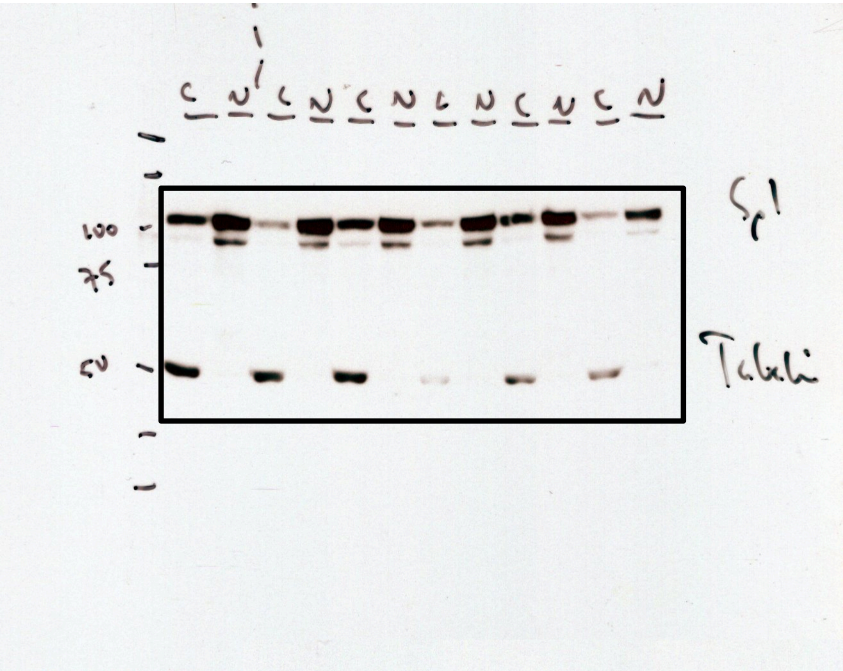

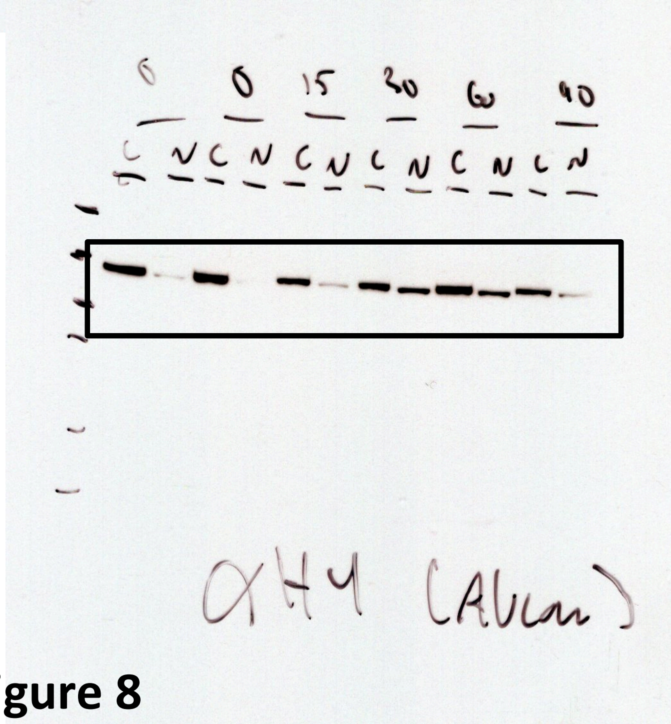

Figure 2A

Supplementary Figure 8

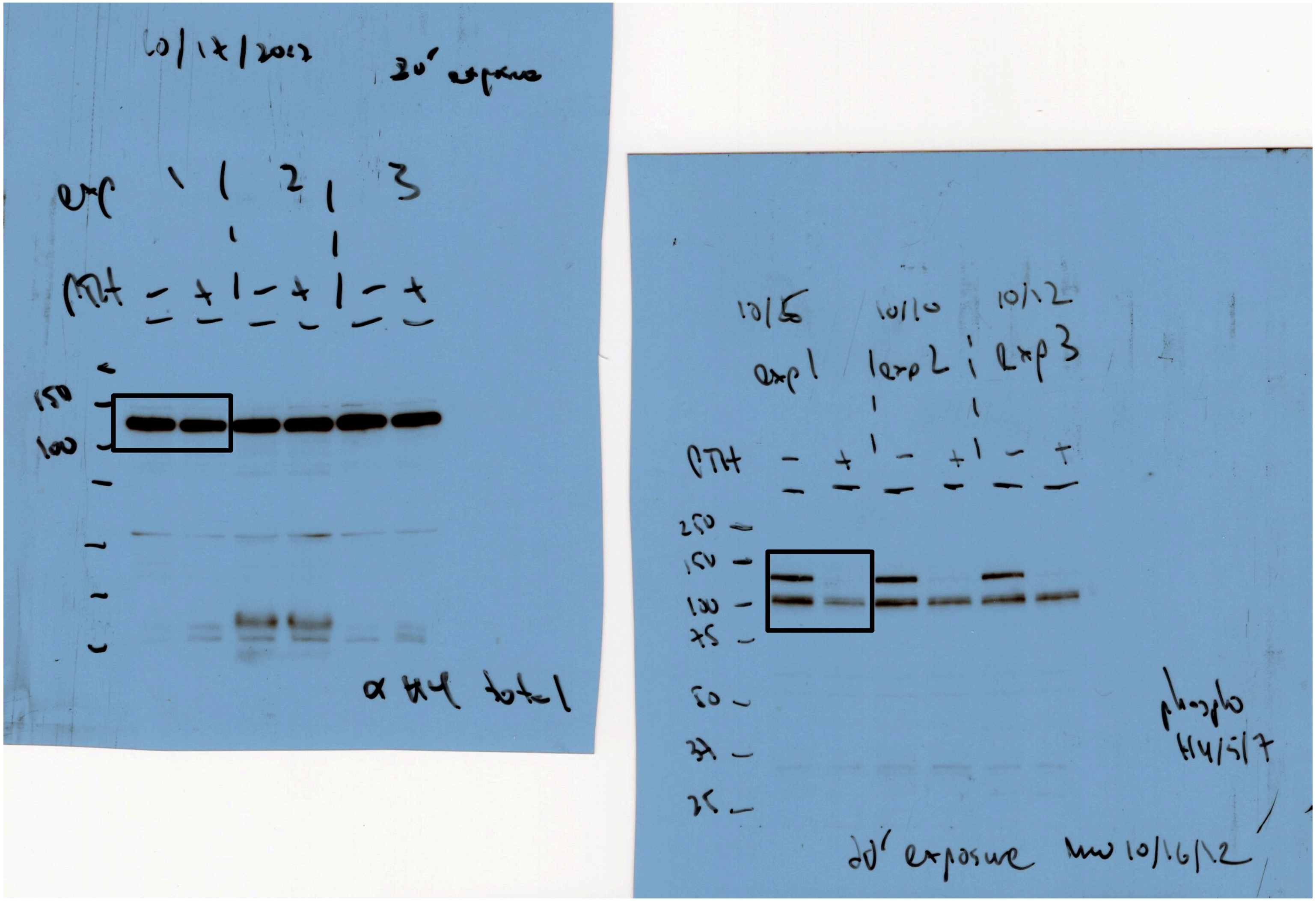

Figure 2B

Supplementary Figure 8

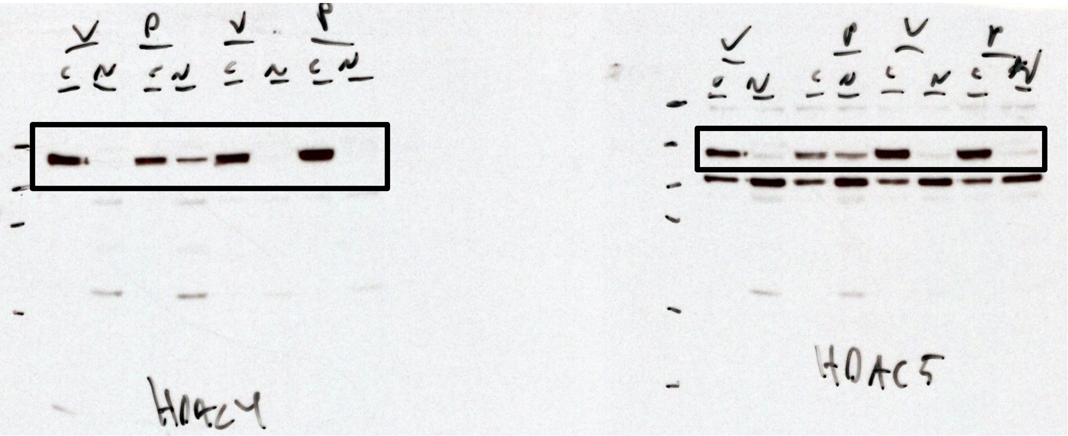

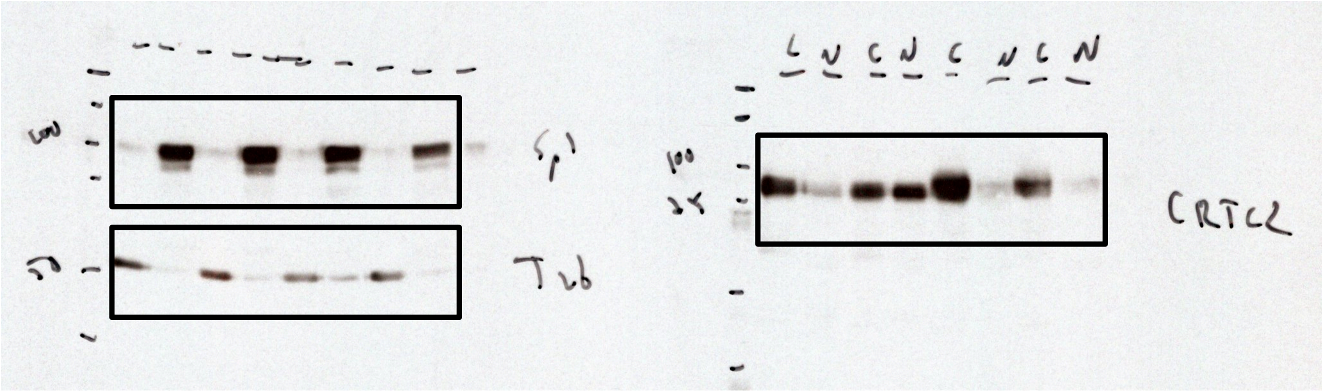

Figure 2C

Supplementary Figure 8

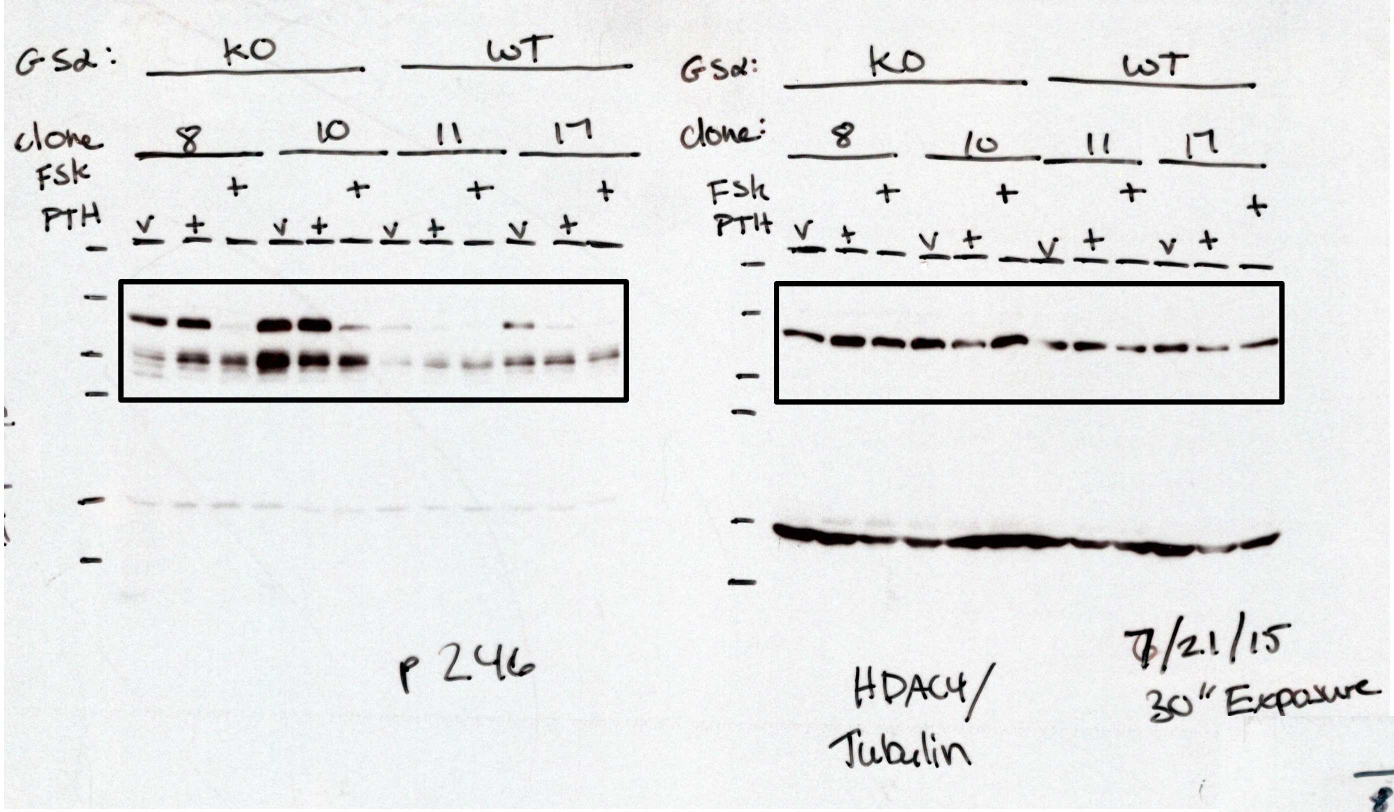

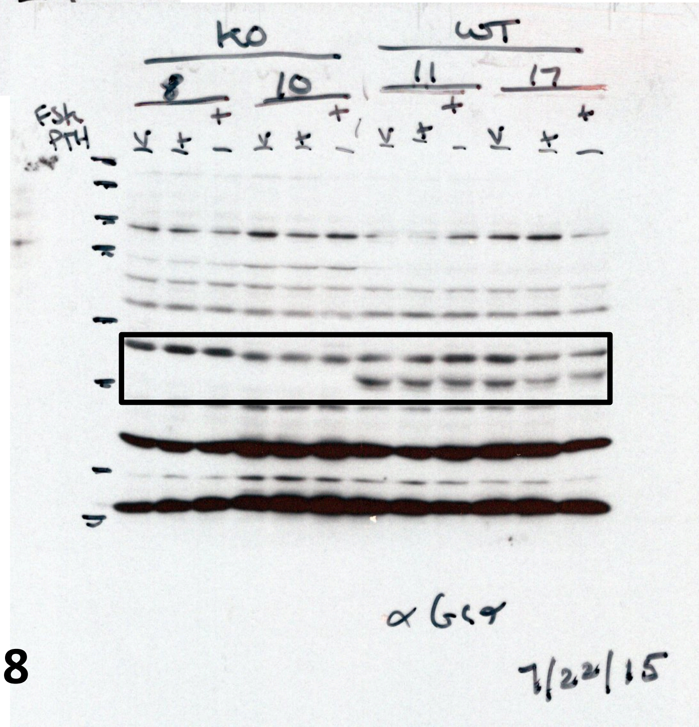

Figure 2D

Supplementary Figure 8

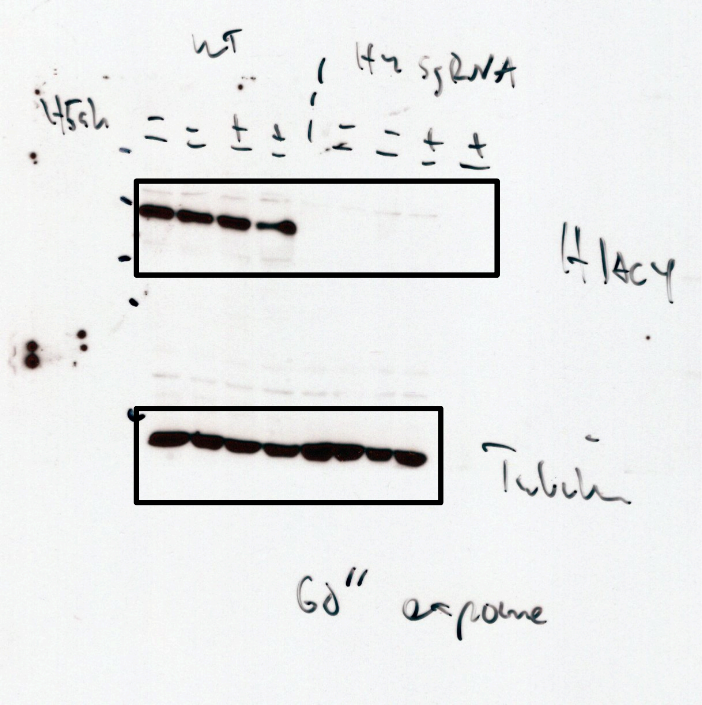

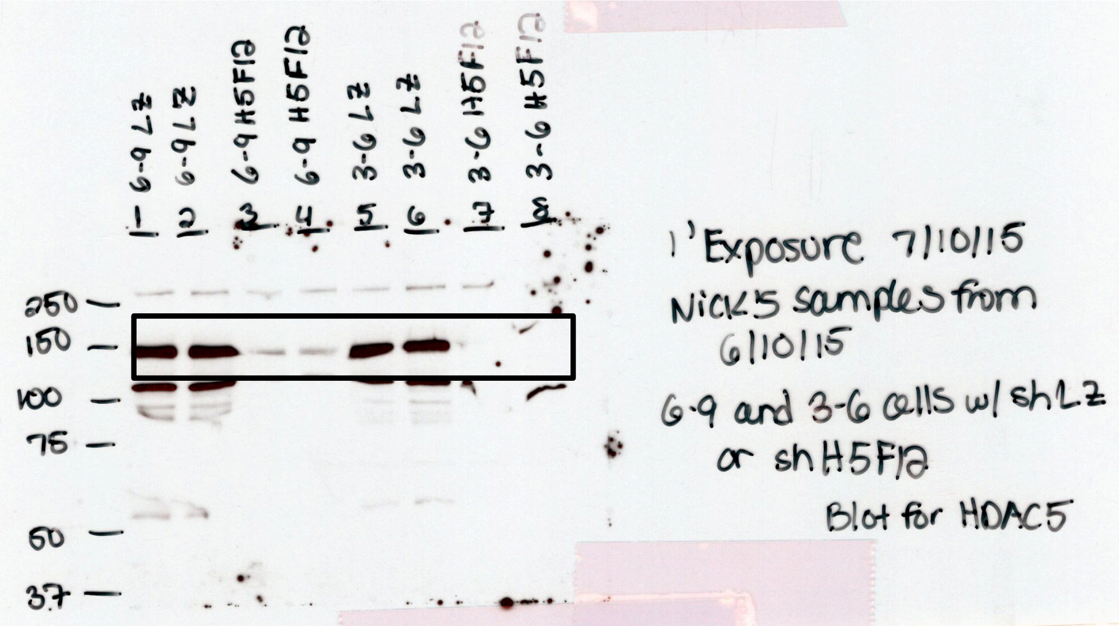

Figure 2E

Supplementary Figure 8

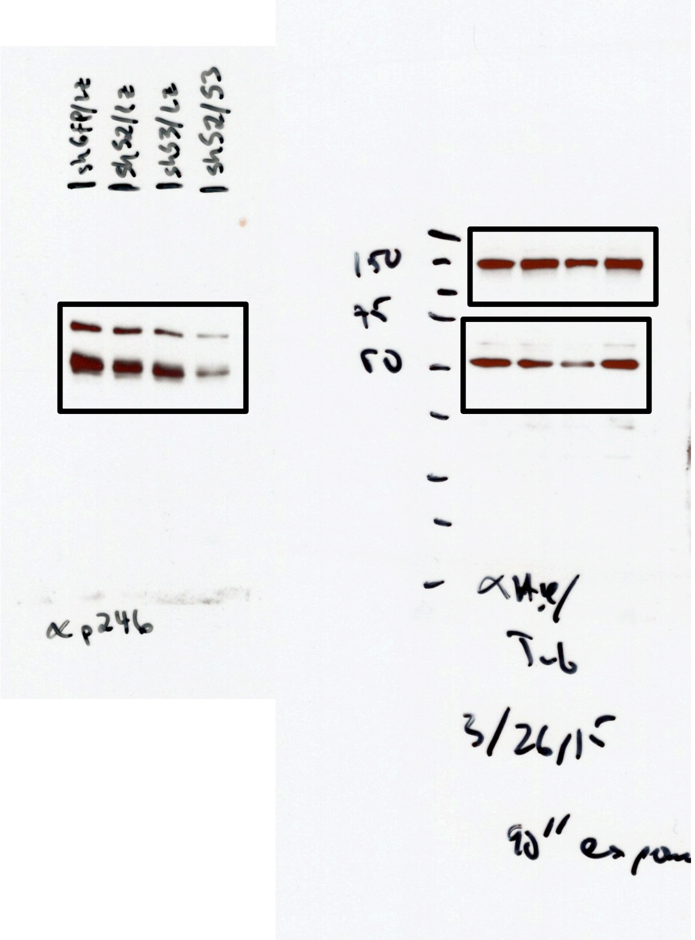

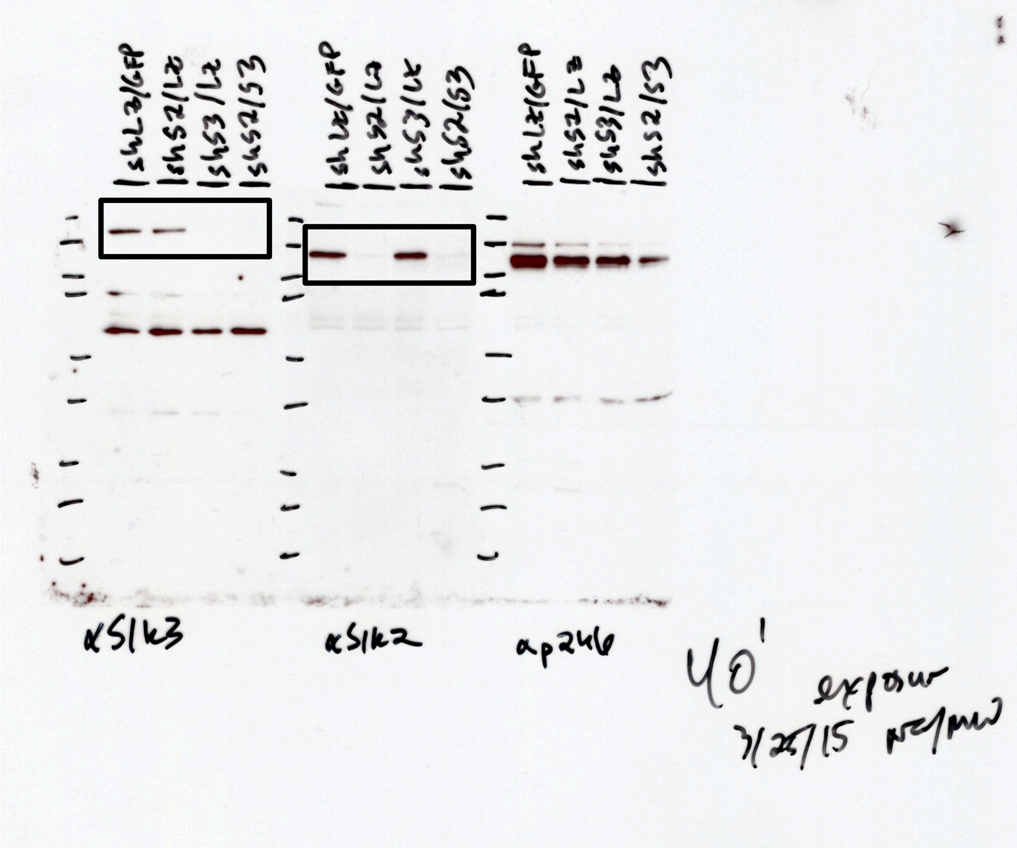

Figure 4A

Supplementary Figure 8

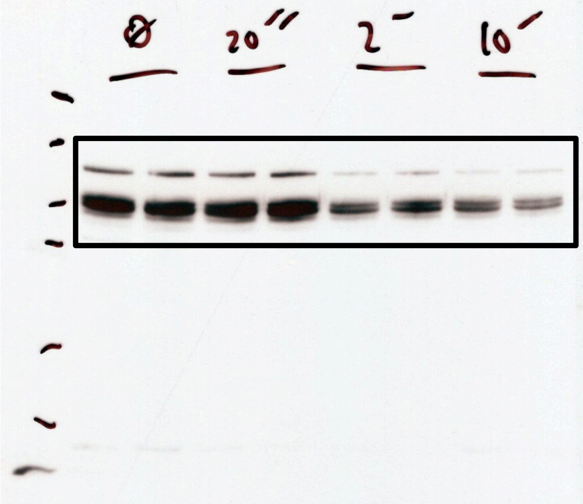

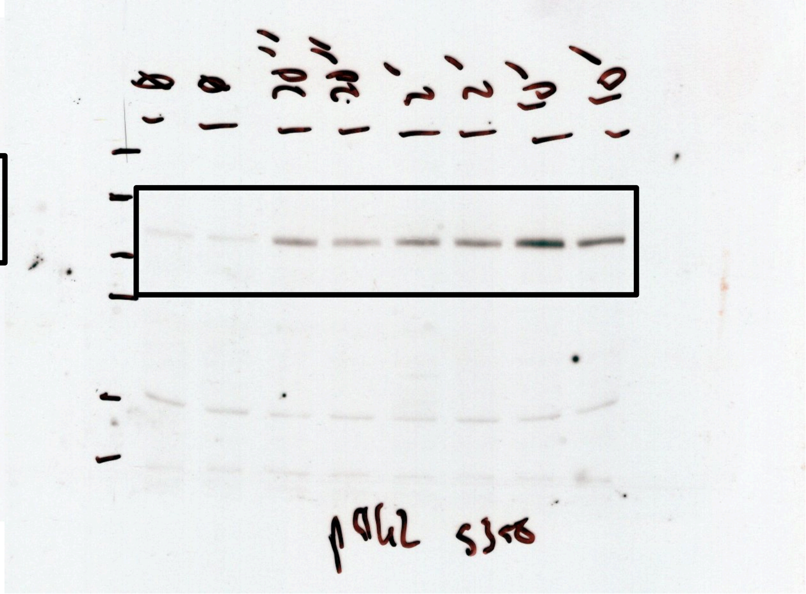

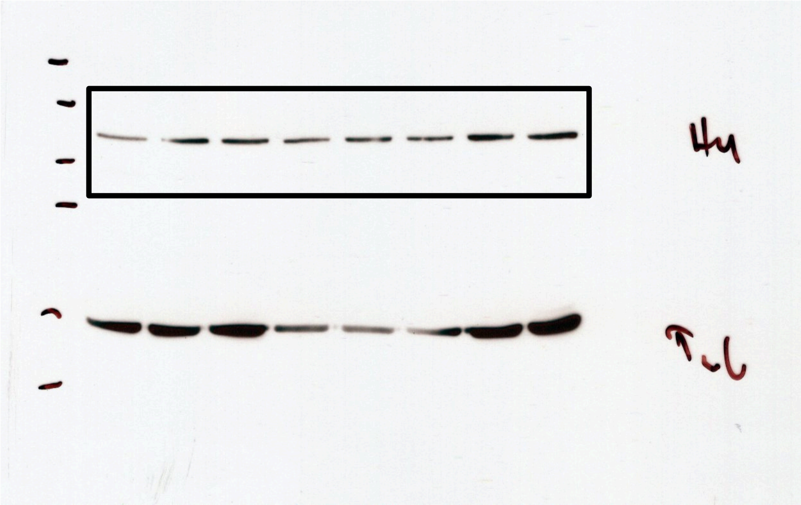

Figure 4C

Supplementary Figure 8

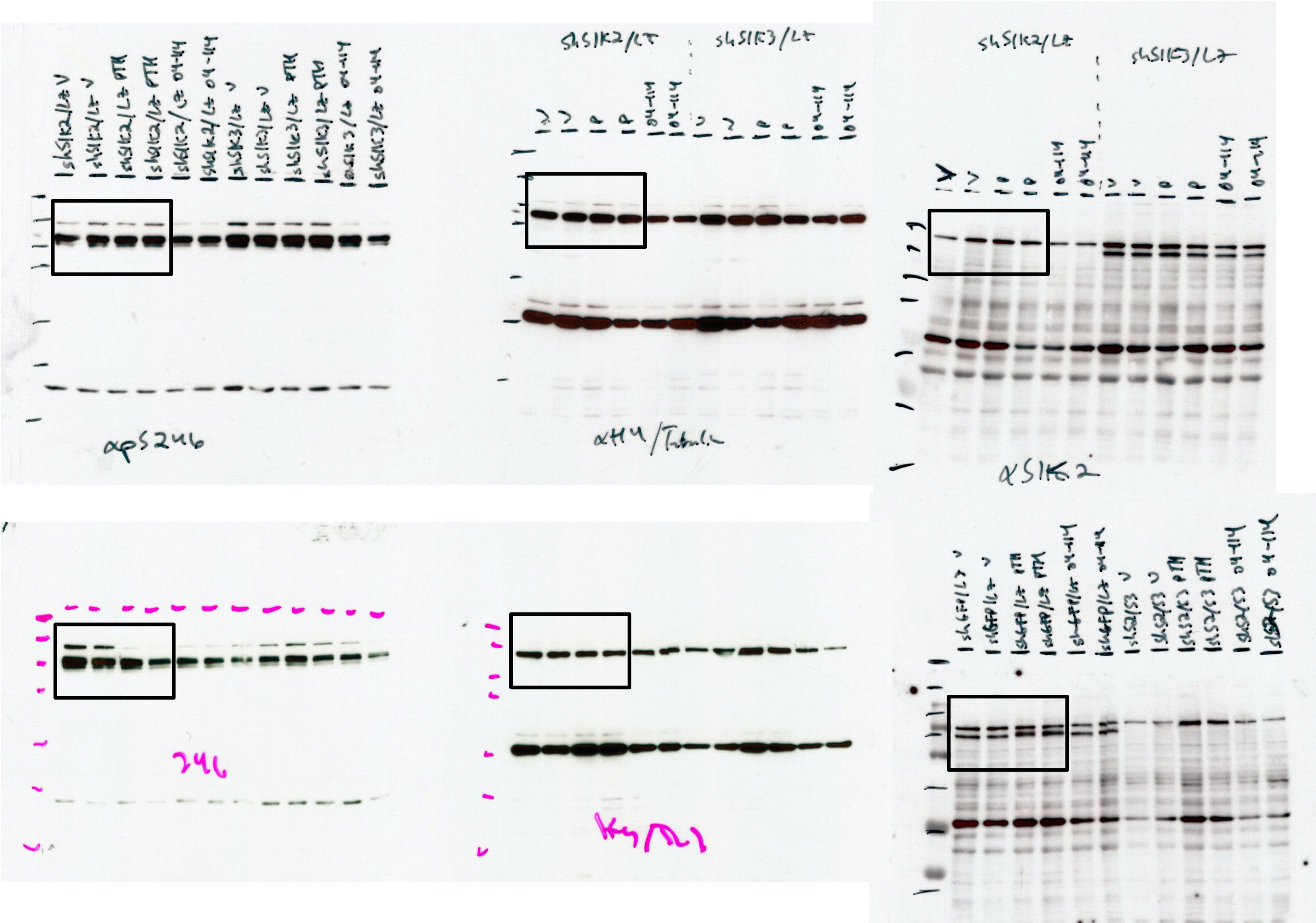

Figure 4E

Supplementary Figure 8

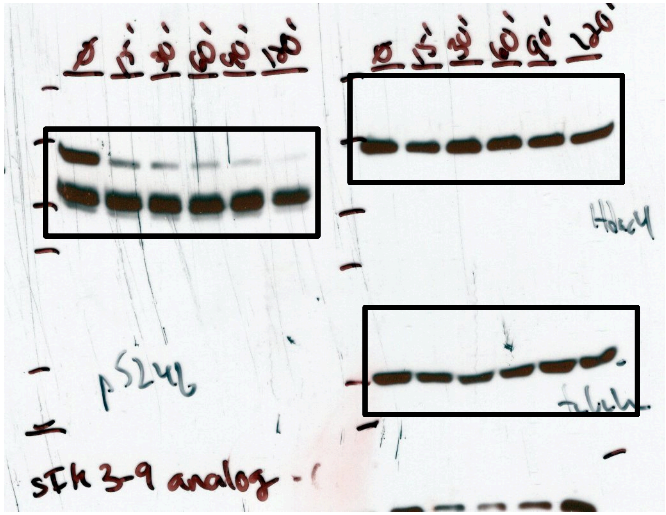

Figure 5B

Supplementary Figure 8

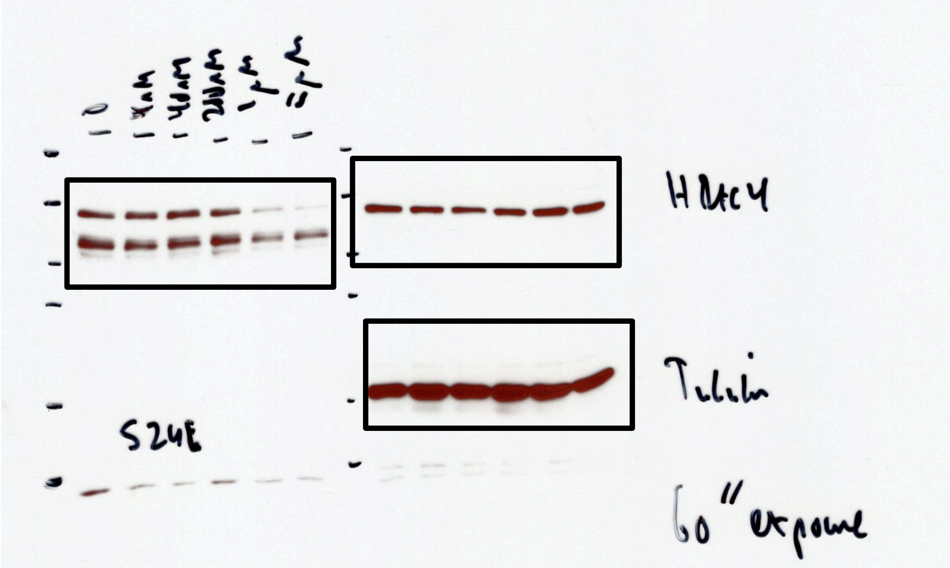

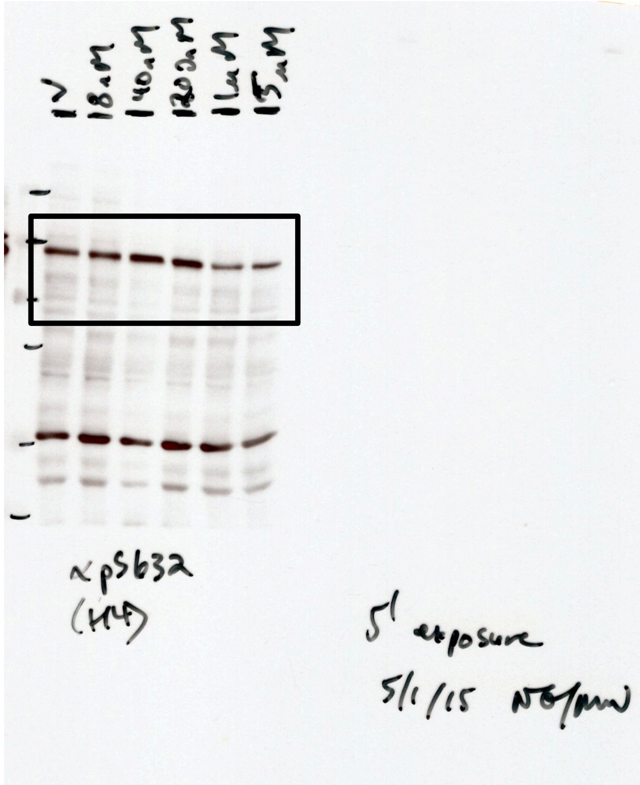

Figure 5C

Supplementary Figure 8

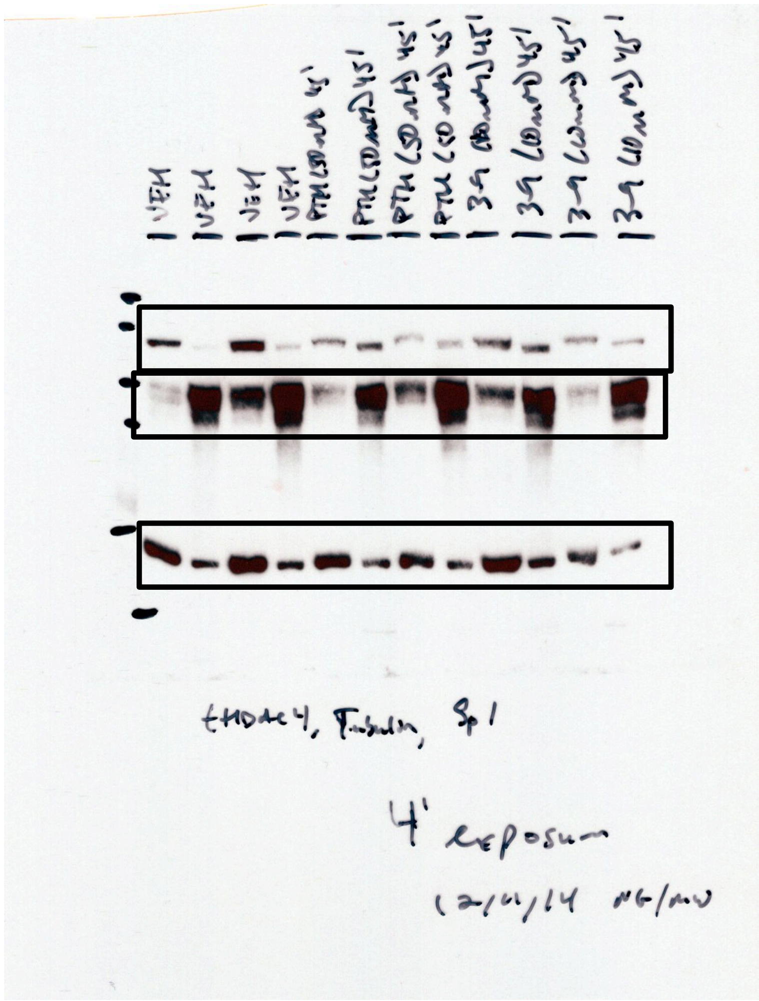

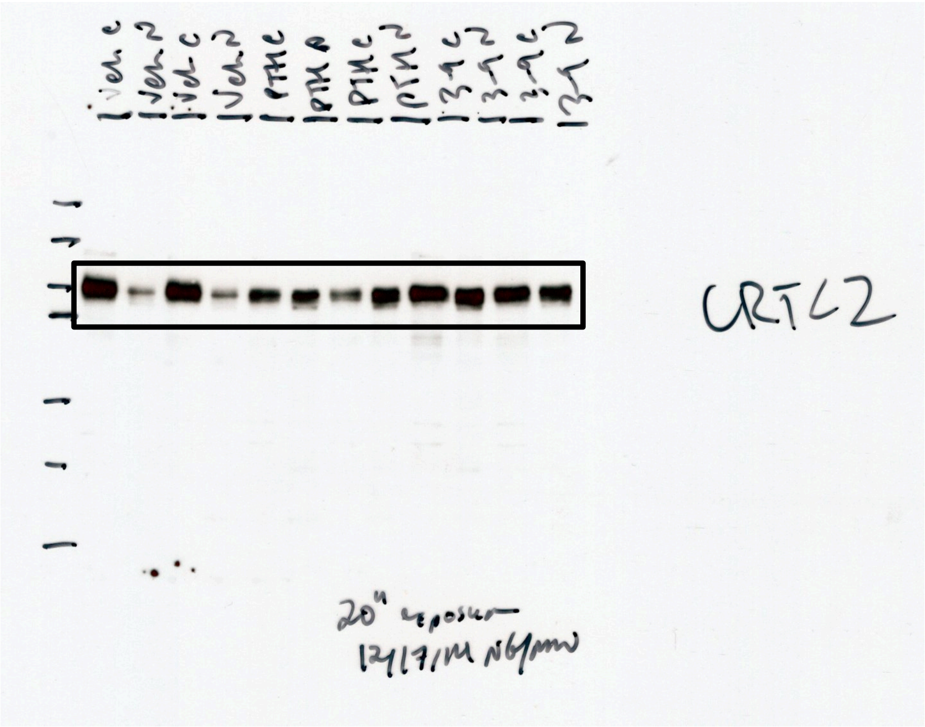

Figure 5D

Supplementary Figure 8

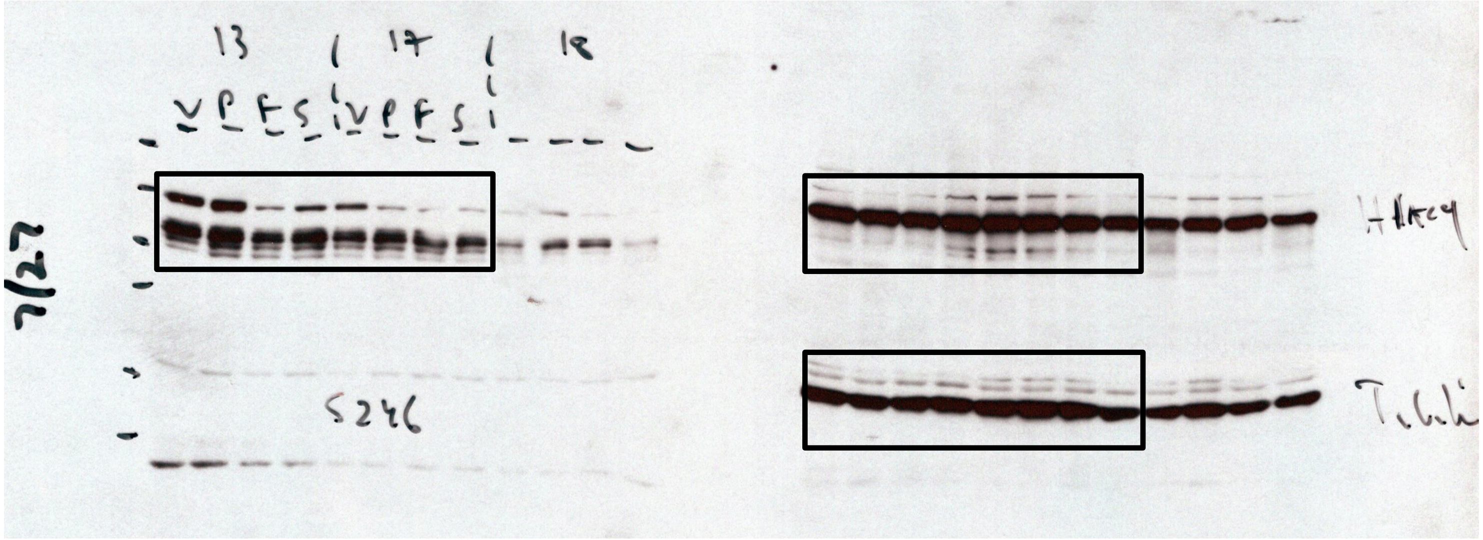

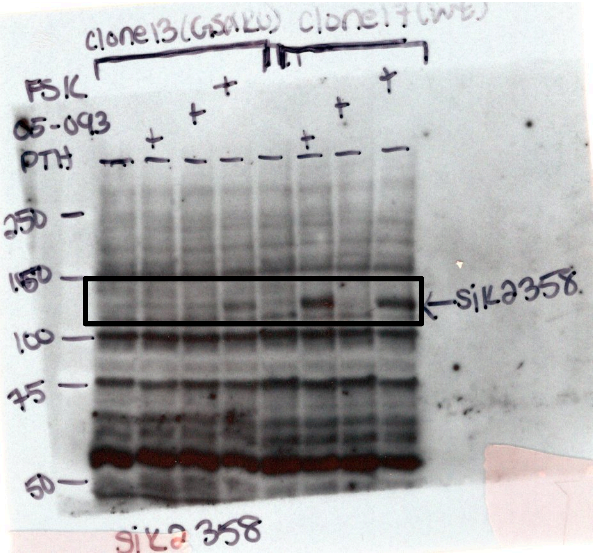

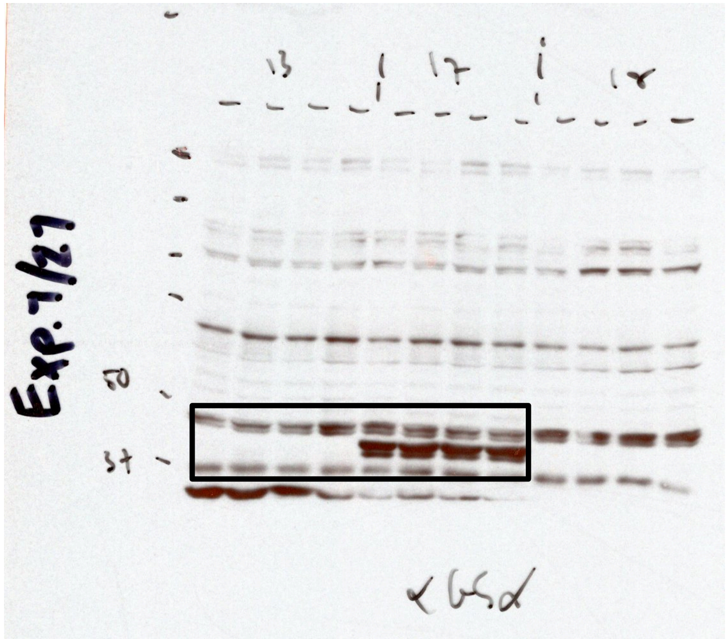

Figure 5D

Supplementary Figure 8

Supplementary Figure 8. Full blots corresponding to portions scanned in main text figures.

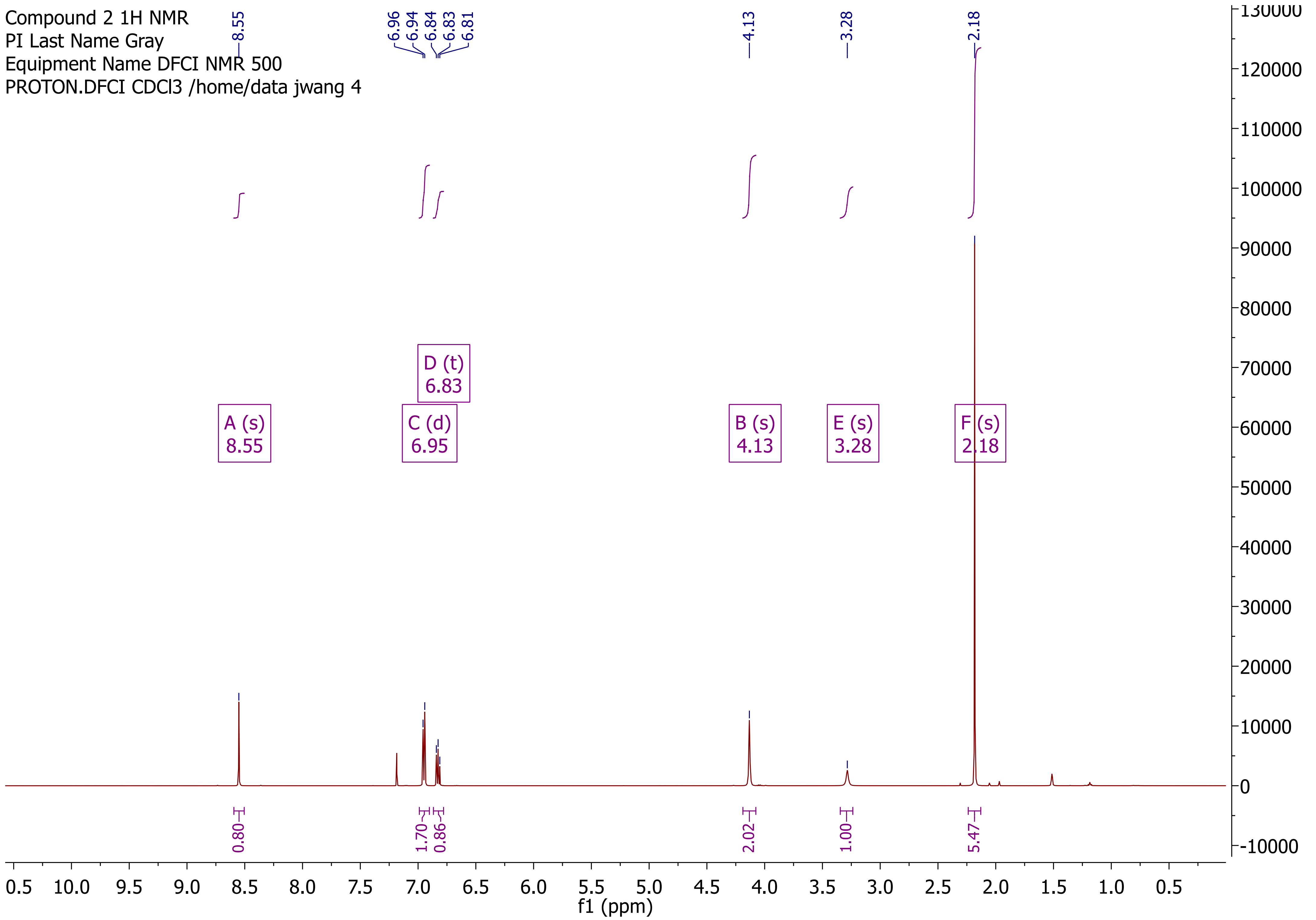

Compound 2 1H NMR
PI Last Name Gray
Equipment Name DFCI NMR 500
PROTON.DFCI CDCl3 /home/data jwang 4
8.55
6.96
6.94
6.84
6.83
6.81
4.13
3.28
2.18
D (t)
6.83
A (s)
8.55
C (d)
6.95
B (s)
4.13
E (s)
3.28
F (s)
2.18
0.80
1.70
0.86
2.02
1.00
5.47
f1 (ppm)

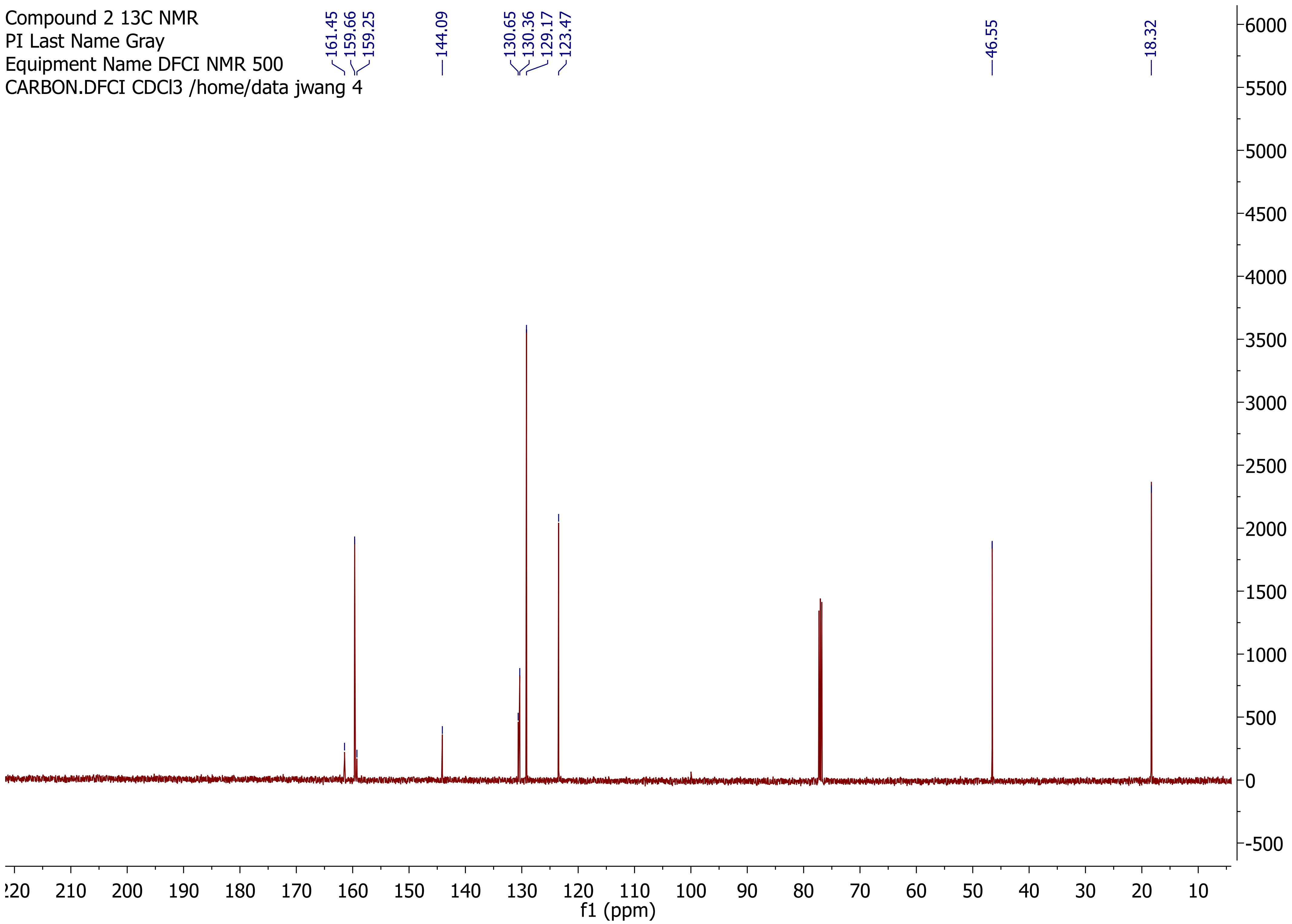
Compound 2 13C NMR
PI Last Name Gray
Equipment Name DFCI NMR 500
CARBON.DFCI CDCl3 /home/data jwang 4
161.45
159.66
159.25
144.09
130.65
130.36
129.17
123.47
46.55
18.32
f1 (ppm)

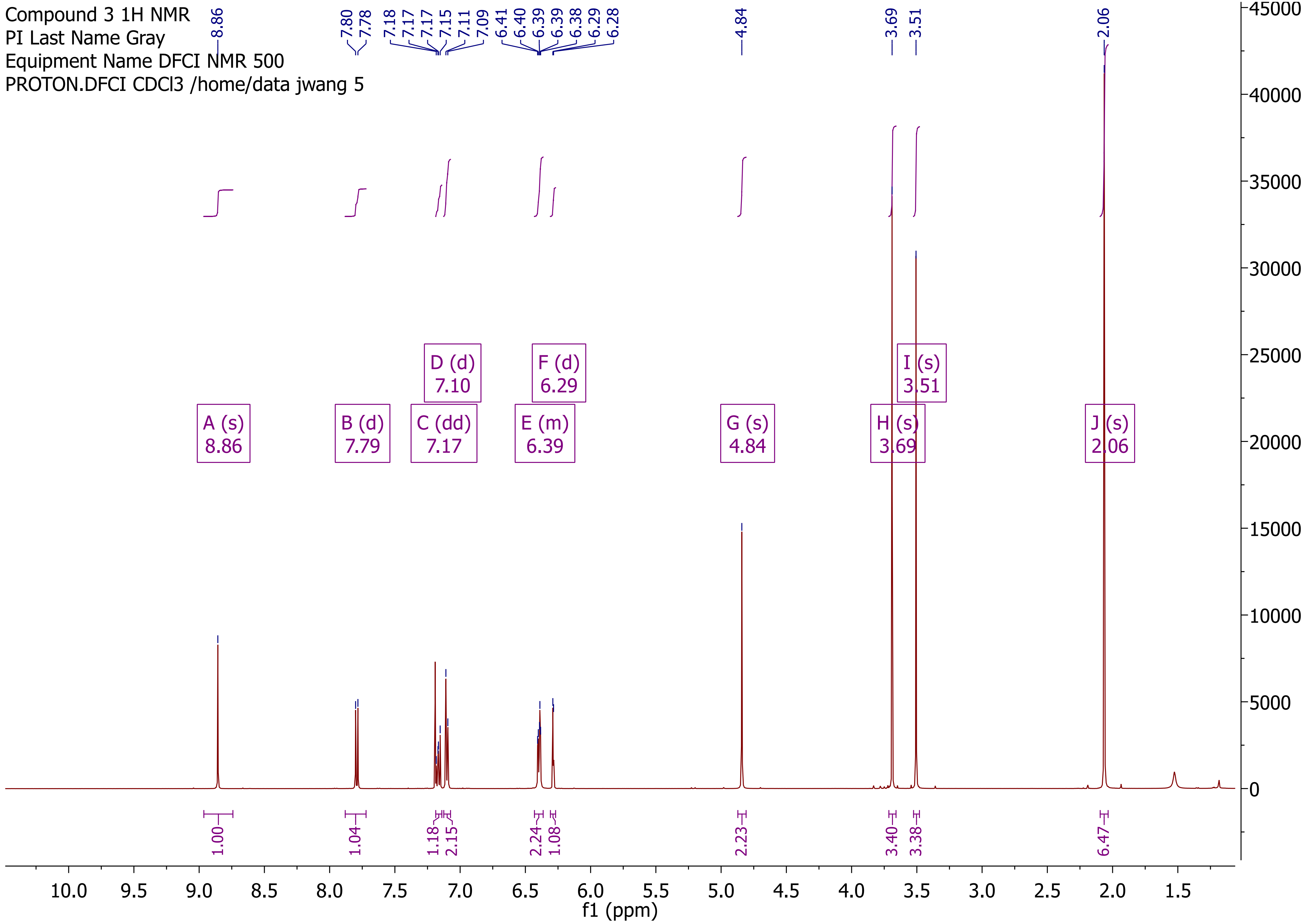
Compound 3 1H NMR
PI Last Name Gray
Equipment Name DFCI NMR 500
PROTON.DFCI CDCl3 /home/data jwang 5
8.86
7.80
7.78
7.18
7.17
7.17
7.15
7.11
7.09
6.41
6.40
6.39
6.39
6.38
6.29
6.28
4.84
3.69
3.51
2.06
A (s) 8.86
B (d) 7.79
C (dd) 7.17
D (d) 7.10
E (m) 6.39
F (d) 6.29
G (s) 4.84
H (s) 3.69
I (s) 3.51
J (s) 2.06
1.00
1.04
1.18
2.15
2.24
1.08
2.23
3.40
3.38
6.47
f1 (ppm)

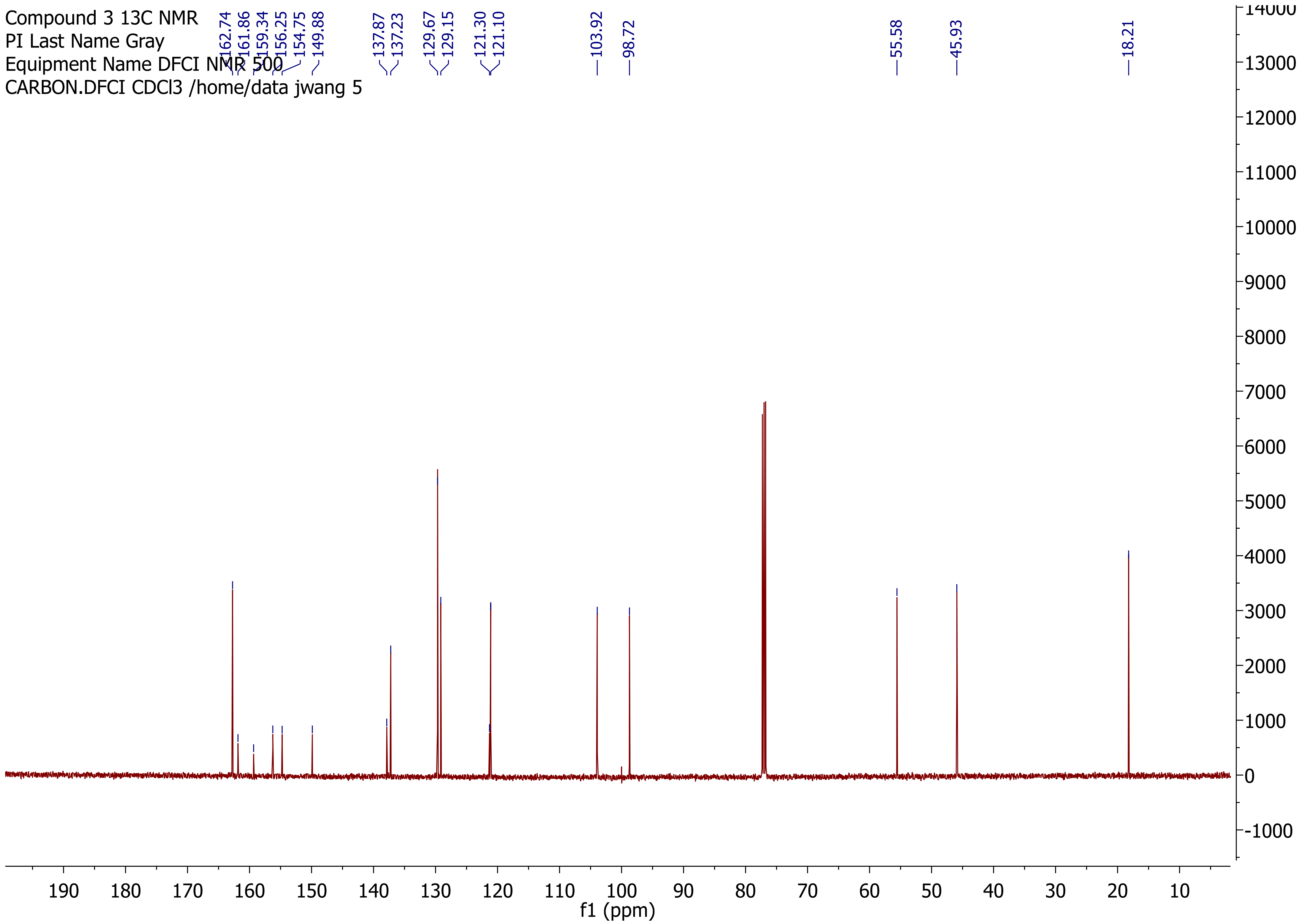
Compound 3 13C NMR
PI Last Name Gray
Equipment Name DFCI NMR 500
CARBON.DFCI CDCl3 /home/data jwang 5
162.74
161.86
159.34
156.25
154.75
149.88
137.87
137.23
129.67
129.15
121.30
121.10
103.92
98.72
55.58
45.93
18.21
f1 (ppm)

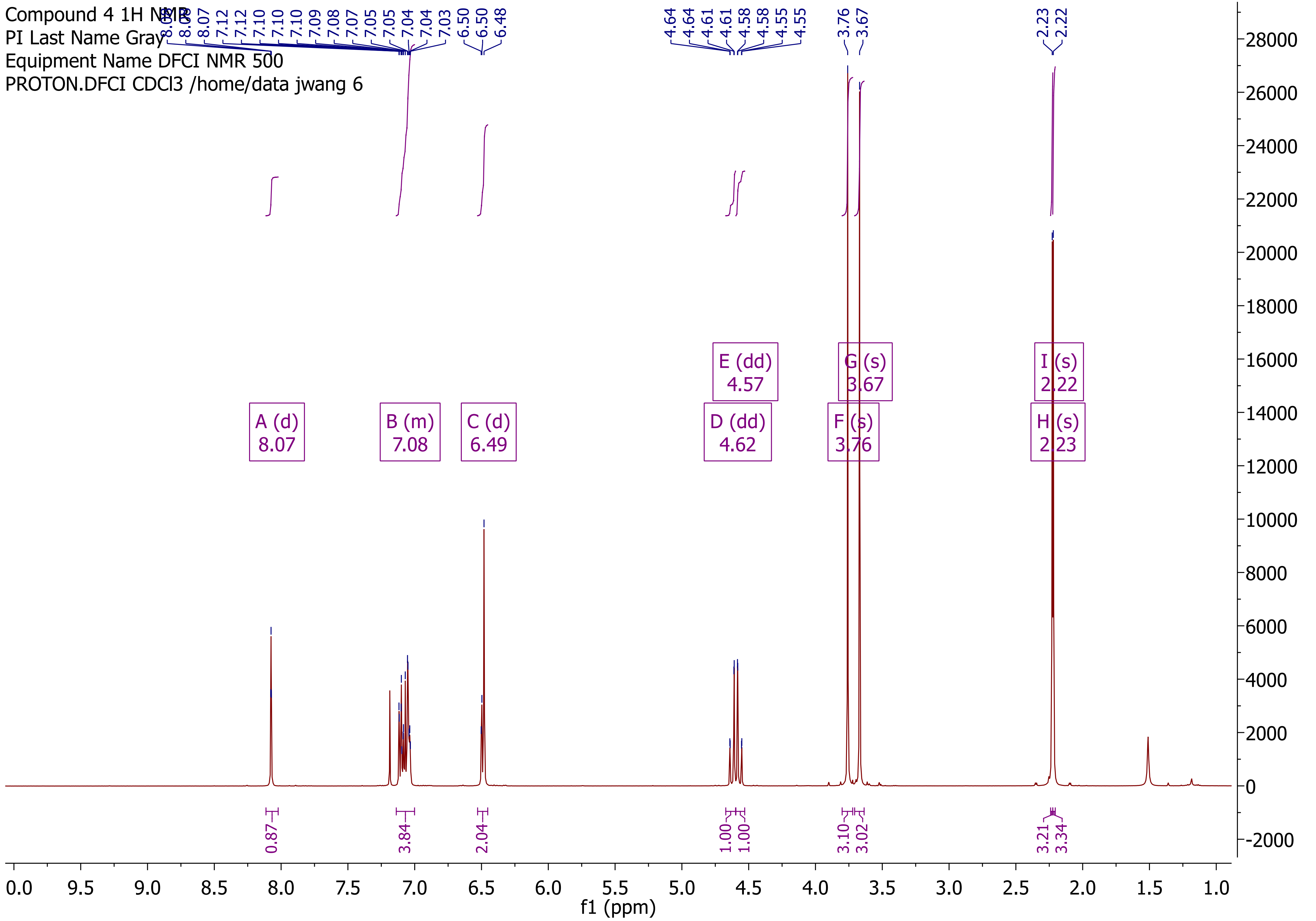
Compound 4 1H NMR
PI Last Name Gray
Equipment Name DFCI NMR 500
PROTON.DFCI CDCl3 /home/data jwang 6
8.07
7.12
7.12
7.10
7.10
7.10
7.09
7.08
7.07
7.05
7.05
7.04
7.04
7.03
6.50
6.50
6.48
4.64
4.64
4.61
4.61
4.58
4.58
4.55
4.55
3.76
3.67
2.23
2.22
A (d) 8.07
B (m) 7.08
C (d) 6.49
D (dd) 4.62
E (dd) 4.57
F (s) 3.76
G (s) 3.67
H (s) 2.23
I (s) 2.22
0.87
3.84
2.04
1.00
1.00
3.10
3.02
3.21
3.34
f1 (ppm)

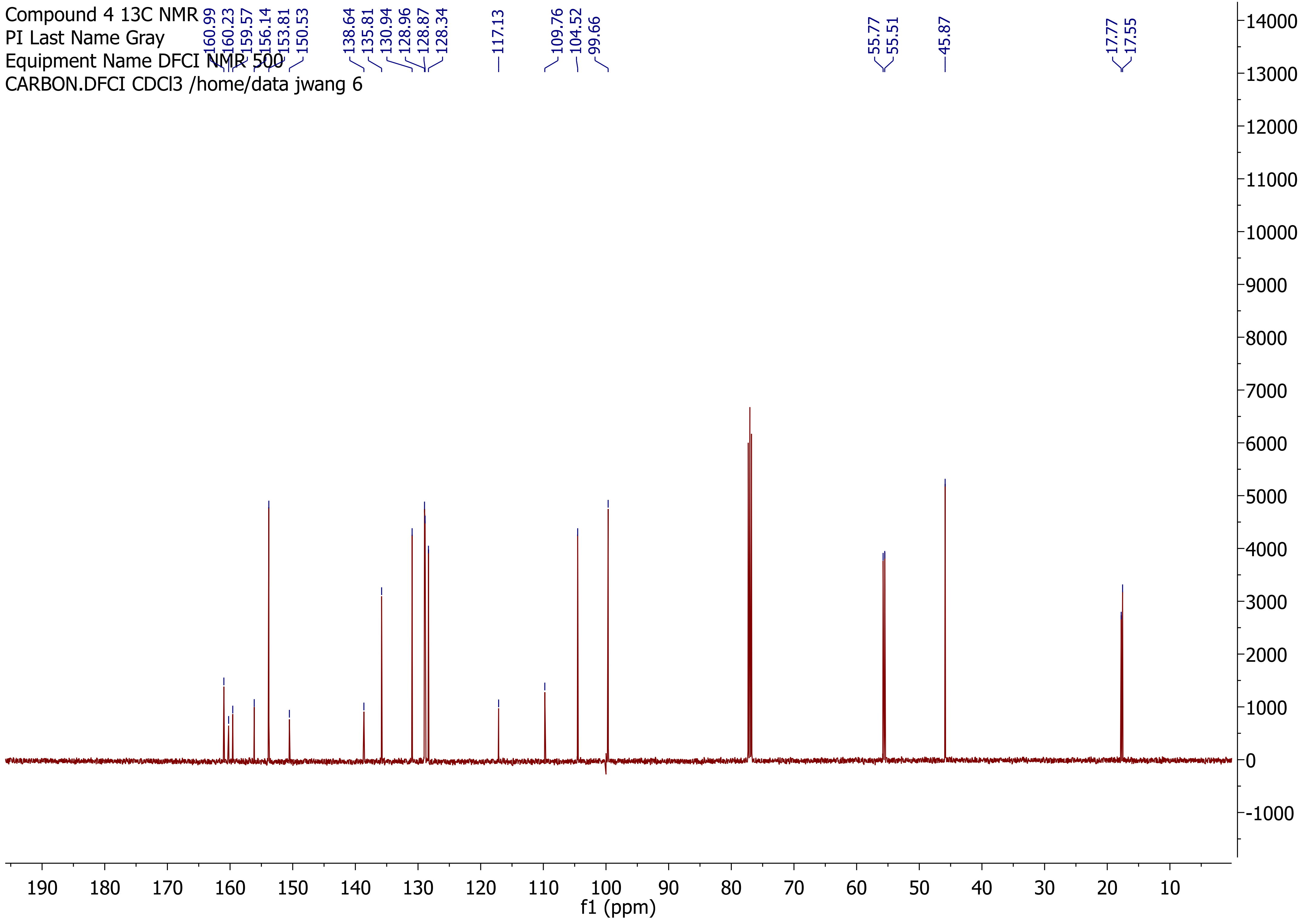
Compound 4 13C NMR
PI Last Name Gray
Equipment Name DFCI NMR 500
CARBON.DFCI CDCl3 /home/data jwang 6
160.99
160.23
159.57
156.14
153.81
150.53
138.64
135.81
130.94
128.96
128.87
128.34
117.13
109.76
104.52
99.66
55.77
55.51
45.87
17.77
17.55
f1 (ppm)

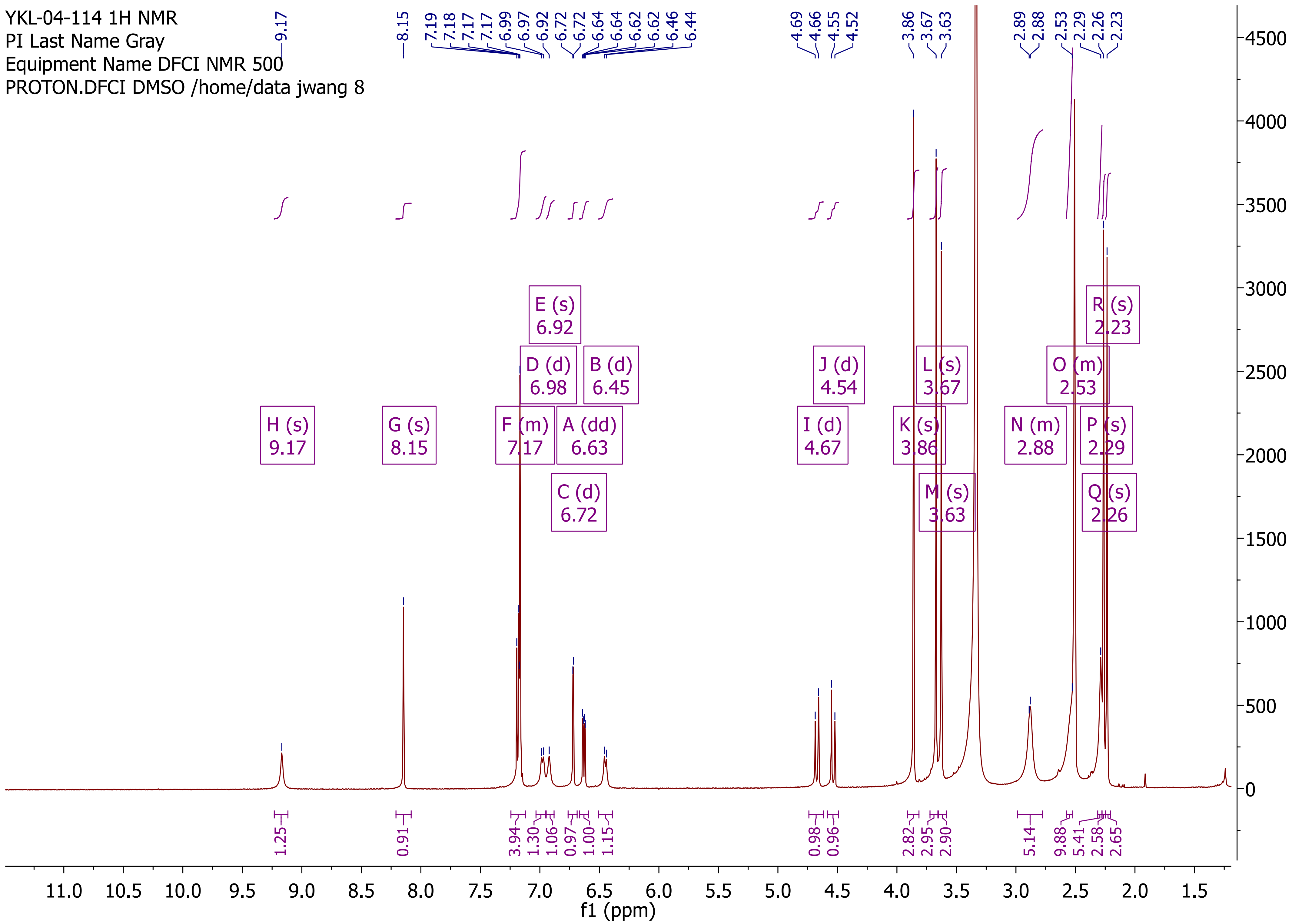
YKL-04-114 1H NMR
PI Last Name Gray
Equipment Name DFCI NMR 500
PROTON.DFCI DMSO /home/data jwang 8
9.17
8.15
7.19 7.18 7.17 7.17 6.99 6.97 6.92 6.72 6.72 6.64 6.64 6.62 6.62 6.46 6.44
4.69 4.66 4.55 4.52
3.86 3.67 3.63
2.89 2.88 2.53 2.29 2.26 2.23
H (s) 9.17
G (s) 8.15
F (m) 7.17
E (s) 6.92
D (d) 6.98
B (d) 6.45
A (dd) 6.63
C (d) 6.72
J (d) 4.54
I (d) 4.67
K (s) 3.86
L (s) 3.67
M (s) 3.63
N (m) 2.88
O (m) 2.53
P (s) 2.29
Q (s) 2.26
R (s) 2.23
1.25
0.91
3.94 1.30 1.06 0.97 1.00 1.15
0.98 0.96
2.82 2.95 2.90
5.14 9.88 5.41 2.58 2.65
f1 (ppm)

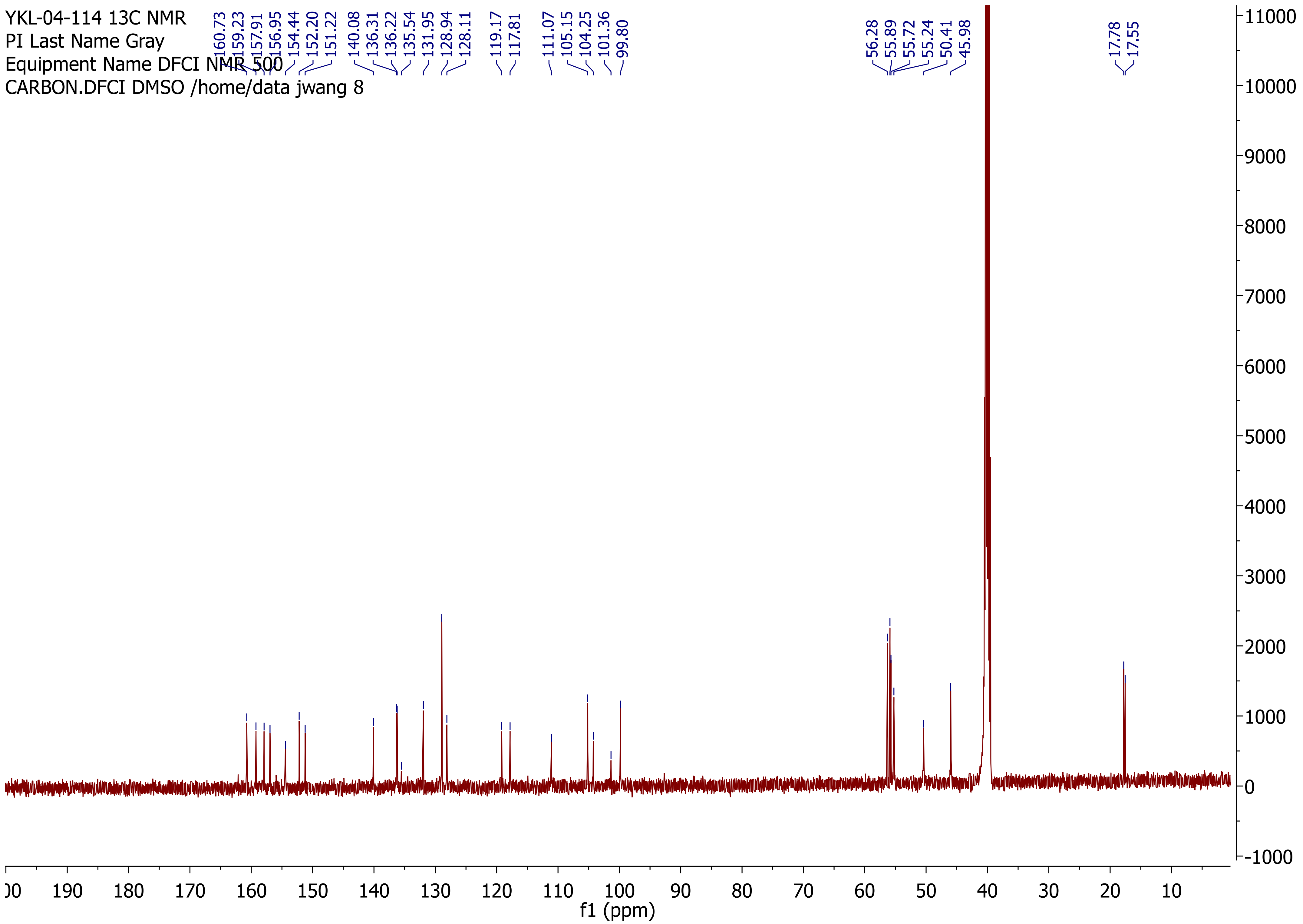

YKL-04-114 13C NMR
PI Last Name Gray
Equipment Name DFCI NMR 500
CARBON.DFCI DMSO /home/data jwang 8
160.73
159.23
157.91
156.95
154.44
152.20
151.22
140.08
136.31
136.22
135.54
131.95
128.94
128.11
119.17
117.81
111.07
105.15
104.25
101.36
99.80
56.28
55.89
55.72
55.24
50.41
45.98
17.78
17.55
f1 (ppm)

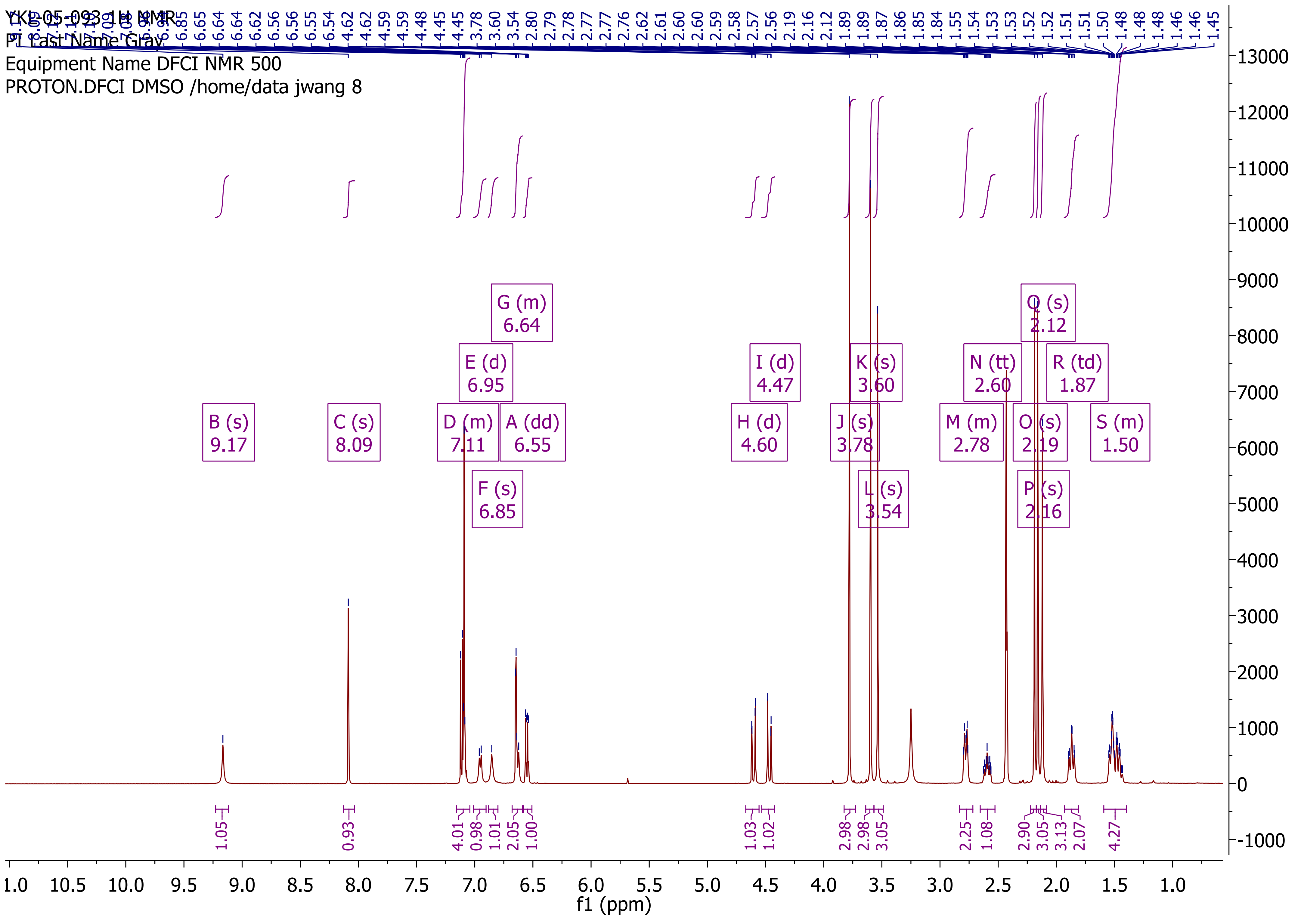
YKL-05-093 1H NMR
PI Last Name Gray
Equipment Name DFCI NMR 500
PROTON.DFCI DMSO /home/data jwang 8
B (s) 9.17
C (s) 8.09
D (m) 7.11
E (d) 6.95
F (s) 6.85
G (m) 6.64
A (dd) 6.55
H (d) 4.60
I (d) 4.47
J (s) 3.78
K (s) 3.60
L (s) 3.54
M (m) 2.78
N (tt) 2.60
O (s) 2.19
P (s) 2.16
Q (s) 2.12
R (td) 1.87
S (m) 1.50
1.05
0.93
4.01
0.98
1.01
2.05
1.00
1.03
1.02
2.98
2.98
3.05
2.25
1.08
2.90
3.05
3.13
2.07
4.27
f1 (ppm)

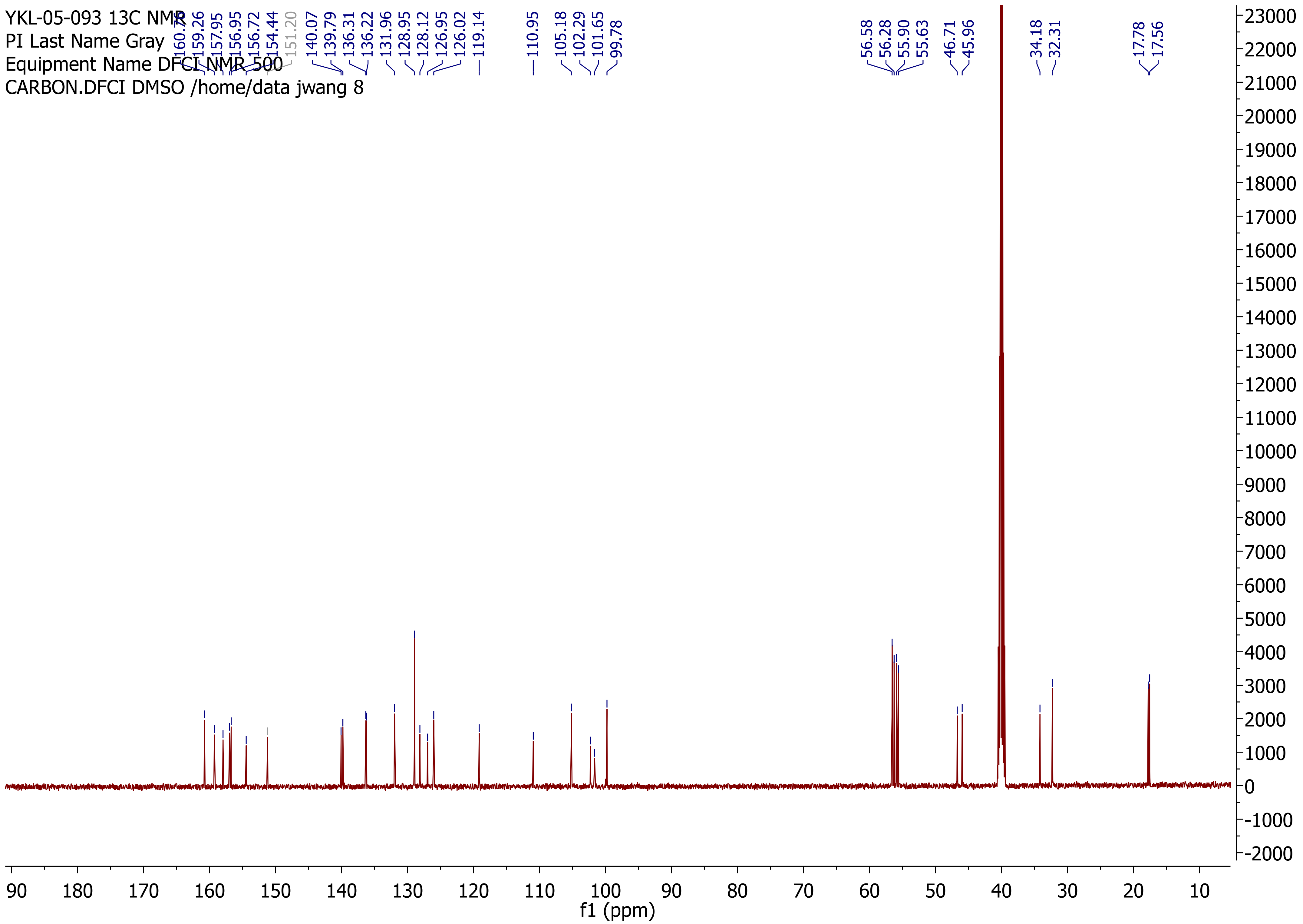
YKL-05-093 13C NMR
PI Last Name Gray
Equipment Name DFCI NMR 500
CARBON.DFCI DMSO /home/data jwang 8
160.7
159.26
157.95
156.95
156.72
154.44
151.20
140.07
139.79
136.31
136.22
131.96
128.95
128.12
126.95
126.02
119.14
110.95
105.18
102.29
101.65
99.78
56.58
56.28
55.90
55.63
46.71
45.96
34.18
32.31
17.78
17.56
23000
22000
21000
20000
19000
18000
17000
16000
15000
14000
13000
12000
11000
10000
9000
8000
7000
6000
5000
4000
3000
2000
1000
0
-1000
-2000
90
180
170
160
150
140
130
120
110
100
90
80
70
60
50
40
30
20
10
f1 (ppm)

Supplementary Figure 9. ^{1}H and ^{13}C NMR data for all compounds tested

	WT (n=8)	HDAC5-/- (n=9)	HDAC4f/f;DMP1-Cre (n=8)	DKO (n=9)
**BV/TV (%)**	8.12±0.88	5.84±0.447	6.72±0.717	**3.45±0.707**
**Tb.Th (um)**	34±1.152	**28.9±1.567**	30.1±1.24	***20.5±0.993***
**Tb.N (/mm)**	2.36±0.201	2.01±0.093	2.2±0.201	1.6±0.263
**Tb.Sp (um)**	414±40.636	487±24.333	464±65.724	**804±163.333**
**MAR (um/day)**	1.96±0.85	**1.25±0.087**	1.74±0.159	**0.96±0.01**
**MS/BS (%)**	50.3±1.177	41.6±2.567	42.8±2.915	**35.4±0.59**
**BFR/BV (%/year)**	2257±56.89	**1462±102.667**	1848±203.534	**1293±66**
**BFR/BS (um3/um2/year)**	361±21.201	**193±23.667**	273±30.035	**125±2.467**
**N.Ob/B.Pm (/mm)**	13.6±.763	**10.4±0.887**	14.1±0.512	11.9±0.813
**Ob.S/B.Pm (%)**	21.1±1.389	**14.2±0.9**	19.6±0.71	**14.9±1.063**
**OS/BS (%)**	14.2±2.039	**7.34±0.84**	14.1±1.852	**3.92±0.623**
**O.Th (um)**	4.04±0.198	3.4±0.173	3.92±0.23	**2.94±0.19**
**N.OC/B/Pm (/mm)**	4.35±0.36	4.87±0.413	5.51±0.332	**6.57±0.79**
**Oc.S/B.Pm %)**	13.2±0.866	14.2±1.07	15.1±0.965	18±2.017
**ES/BS (%)**	3.3±0.44	2.99±0.217	4.26±0.431	3.54±0.65

Supplementary Table 1. Tibial histomorphometry results for 8 week old female mice of the indicated genotype.
For each parameter, the value is shown followed by s.e.m.
Statistical analysis was performed by one-way ANOVA followed by Tukey's posthoc t test.
Values in **bold** indicate p<0.05 comparing WT and the strain of interest.
Values in *italics* indicates p<0.05 comparing HDAC5-/- and DKO groups.

Supplementary Table 2: Results of YKL-05-093 profiling against 96 recombinant kinases

Gene Symbol	% of control activity at 71 nM
ABL1	0.2
ACVR1B	50
ADCK3	93
AKT1	92
ALK	23
AMPK-alpha1	94
ARK5	77
AURKA	12
AXL	68
BMPR2	87
BRAF	66
BRSK1	74
BTK	0.45
CDK11	88
CDK2	99
CDK3	80
CDK7	85
CDK9	88
CHEK1	42
CSF1R	0.85
CSNK1D	98
CSNK1G2	100
DCAMKL1	89
DYRK1B	93
EGFR	5.9
EPHA2	7.1
ERBB2	3.5
ERBB4	6.1
ERK1	100
FAK	90
FGFR2	8.9
FGFR3	24
FLT3	20
GSK3B	68
HCK	1.2
HIPK2	100
IGF1R	100
IKK-alpha	93
IKK-beta	84
INSR	56

JAK2	13
JNK1	97
JNK2	89
KIT	1.8
LCK	0.75
LKB1	85
MAP3K4	60
MAPKAPK2	85
MARK1	84
MARK3	40
MEK1	20
MEK2	12
MELK	36
MET	92
MKNK1	76
MLK1	12
p38-alpha	0.95
p38-beta	47
PAK1	6.6
PAK2	75
PAK4	88
PCTK1	91
PDGFRA	11
PDGFRB	0.35
PDPK1	55
PIK3C2B	100
PIK3CA	84
PIK3CG	80
PIM1	89
PIM2	97
PIM3	84
PKAC-alpha	100
PLK1	100
PLK3	90
PRKCE	92
**QSK**	**5.3**
RAF1	100
RET	3.6
RIOK2	77
RIPK2	16
ROCK2	70
RSK2	51
**SIK**	**0.6**

**SIK2**	**10**
SNARK	6
SRC	0
SRPK3	88
TGFBR1	90
TIE2	32
TRKA	74
TSSK1B	83
TYK2	49
ULK2	31
VEGFR2	26
YANK3	95
ZAP70	52

Supplementary Table 3.
Differentially-expressed genes: >2-fold, FDR<0.05.

Up with PTH only	Up with YKL-05-093 alone	Up with both PTH and YKL-05-093	Down with PTH only	Down with YKL-05-093 only	Down with both PTH and YKL-05-093
2310043M15Rik	1700023H06Rik	Ackr3	1700001L05Rik	Arc	6330403L08Rik
Abtb1	1700023L04Rik	Acsl3	2310022B05Rik	Arrdc4	Adra1d
Aldh3a1	A930018M24Rik	Adrb2	2700038G22Rik	Atoh8	Cd200
Ankrd44	Adamts1	Alx3	9930013L23Rik	Bahcc1	Chst15
Arl4c	Adck3	Arl4d	Abi2	Bcl2	Cxcl12
Baalc	Adrb1	Arrdc3	Adamts18	Ccl2	Cyp26b1
Batf	Aim1	Avpi1	Ahrr	Ccl7	Dlk2
Bglap	Apbb1	BB557941	Akap6	Csf1	Egr2
Bglap2	AU021092	C2cd4c	Ankrd34a	Ctgf	Enc1
C1qtnf1	Bmf	Cebpd	Ano6	Dlx2	Esm1
Camk4	Bmp6	Col13a1	Apln	Dlx5	F3
Ccdc109b	Btbd17	Crem	Bcar3	Dlx6	Fam198b
Ccdc152	C130050O18Rik	Crispld2	Bmp2	Dmp1	Fjx1
Cda	Cd24a	Cxcl1	Bok	Dusp4	Fzd5
Cebpb	Col11a2	Enpp6	Car8	Dusp6	Fzd8
Ch25h	Dhrs3	Eya2	Cd2ap	Egr1	Gm10715
Chst12	Dusp1	Fam167a	Cdk5r1	Eps8	Gm10717
Cited1	Fbxo32	Fam20a	Cdo1	Etv5	Gm10718
Clec2d	Fosl2	Fos	Chst3	Gcnt4	Gm10800
Col2a1	Gm11837	Gadd45a	Chsy1	Gm11168	Gm10801
Cyp1b1	Gm22314	Gja1	Cmya5	Gm16516	Gm13186
Cyp26a1	Gm22633	Glis1	Deptor	Gm23296	Gm13493
Ddc	Gm24119	Gm22220	Dixdc1	Gm25047	Gm21738
Dio3	Gm25395	Gm22288	Dlx3	Gm26982	Gm26507
Dio3os	Gm9949	Gm22421	Dtx4	Gm3200	Gm26870
Dnajc12	Gpr133	Gm22623	Dusp7	Gm9987	Gm5763
Dpt	Gprc5c	Gm22628	Eepd1	Hey1	Gpr176
Efnb2	Grhl3	Gm23287	Ell2	Hmga2	Gprin3
Emb	Hgf	Gm23445	Fam101b	Hoxc12	Hdac9
Fam134b	Hrc	Gm23927	Fam102a	Hoxc13	Id3
Fam169b	Inhbb	Gm23947	Fam13c	Id1	Il17rd
Fam198a	Kctd7	Gm23966	Fam180a	Id2	Klf5
Fam69c	Kdr	Gm23971	Fam217b	Irf5	Lfng
Fas	Krt80	Gm24204	Fam43a	Klhdc8a	Lmcd1
Fbxo31	Mn1	Gm24316	Farp2	Krtap1-5	Nuak1
Flrt2	Ncf1	Gm24447	Fgd3	Mical2	Pdgfa
Foxf1	Nfil3	Gm24620	Fhod1	Nfkbie	Rasl11a
Fxyd5	Nr4a3	Gm24917	Foxd1	Pcdh10	Rgs3
Fzd1	Pdzrn3	Gm24968	Gadd45g	Pdp1	Shisa2
Gfra1	Per1	Gm25101	Gcnt1	Prkg2	Spry1
Gm10327	Plekha5	Gm25514	Gli1	Ptprj	Tbx2
Gm10638	Ptgfr	Gm25682	Gm10136	Rin1	Thbs1
Gm13705	Rftn1	Gm26072	Gm10602	Rnf150	Tiam2
Gm16062	Rhpn2	Gm26323	Gm10722	Rspo3	Tmem229b
Gm22265	Serpinb6b	Gm26324	Gm11944	Sacs	Vgll3
Gm22307	Sik1	Gm26331	Gm129	Serpina3f	
Gm22488	Sox4	Gng4	Gm15663	Serpina3g	
Gm22513	Spon2	Has2	Gm16185	Skil	
Gm22661	Ston2	Hdac4	Gm17045	Smad7	
Gm22980	Tcp11l2	Igf1	Gm17275	Smad9	
Gm22997	Tmie	Il1rl1	Gm20471	Snai2	
Gm23008	Usp2	Il6	Gm20655	Socs1	
Gm23137	Utp14b	Kcne4	Gm23388	Socs5	
Gm23140	Wnt7b	Kcnj2	Gm6478	Spred1	
Gm23143	Xylt1	Kcnk10	Gm6578	Spry4	
Gm23153	Ypel1	Limch1	Gm9869	Synj2	
Gm23201		Lpcat2	Hoxa4	Tmem2	
Gm23240		Lrrc17	Hps3	Tnfaip3	
Gm23511		Mcam	Hspb7	Tnfrsf12a	
Gm23523		Metazoa_SRP	Ihh		
Gm23686		Mrgprf	Inhba		
Gm24207		mt-Co3	Insc		
Gm24299		mt-Tl1	Irx1		
Gm24305		mt-Tm	Irx3		
Gm24317		N4bp2l1	Irx5		
Gm24407		Ncald	Kcnb1		
Gm24438		Nr4a1	KCTD12		
Gm24449		Nr4a2	Kif21b		
Gm24494		Nrp1	Klf4		
Gm24596		Pde4b	Klhl30		
Gm25099		Pde4d	Krt12		
Gm25107		Phex	Lbh		
Gm25135		Pim1	Lifr		
Gm25189		Plau	Lmo7		
Gm25327		Prex1	Lmod1		
Gm25380		Rasl10b	Lpar3		
Gm25414		Rgs2	Lyst		
Gm25681		Rnf122	Mars2		
Gm25739		Rprl2	Mef2c		
Gm25781		S1pr1	Mgat5		
Gm25793		Scg2	Mtus2		
Gm25970		Serpinb1a	Murc		
Gm26104		Shc2	Ndnf		
Gm26107		Slc7a7	Neurl2		
Gm26202		Slpi	Nexn		

Gm6872
Gm9889
Got1
Gpr153
Grem2
Hopx
Ifngr1
Il4ra
Irak3
Itga11
Itga9
Itgb3
Kremen1
Krt31
Krt33b
Ksr1
Lef1
Lif
Lrp8
Ly6a
Ly6c1
Megf10
Mgp
Mir3068
Mir5136
Mir677
Mmp13
mt-Atp6
mt-Tv
mt-Tw
Nap1l5
Net1
Notum
Osmr
Parvb
Pdk4
Pdpn
Pgpep1l
Pitpnc1
Pkdcc
Plaur
Plxna2
Ppap2b
Ppfibp2
Prr5
Ptp4a1
Rnu12
Rnu73b
Rny3
Rprl3
Scarna17
Sfrp1
Sfrp4
Slc1a3
Slc37a2
Slc43a2
Smim3
Snora28
Snora36b
Snora47
Snora62
Snord100
Snord104
Snord110
Snord19
Snord35b
Snord49a
Snord49b
Snord61
Snord65
Snord71
Snord82
Snord85
Soga2
Stat3
Tgfa
Tll1
Tnfaip6
Tnfrsf19
Vit
Wdr45
Wif1
Wisp2
Wnt9a
Zfp52
Zfp791
Zhx2

Snai1
Snora15
Tmem100
Tnfrsf9
Tnfsf11
Trib2
Trp53inp1
Tsc22d3
Tspan11
Vdr
Wnt4
Ypel3

Nhsl1
P2rx5
Pak3
Panx3
Pawr
Phospho1
Pitx2
Polr3e
Pparg
Ppm1e
Rassf3
Rcan2
Rcor2
Rnf144b
Rnf43
Rpgrip1l
Rtn4rl1
Runx1
Runx2
Sap30
Satb2
Scn3a
Serpine1
Slc22a23
Slc25a13
Slc40a1
Slc8a3
Smpd3
Smtnl2
Sncaip
Snta1
Snx7
Sp7
Stc2
Swap70
Tbc1d4
Tcf7
Tmeff1
Tmem119
Tmtc2
Tnfrsf11b
Tnik
Trmt61a
Wisp1
Wnt10b
Zbtb16
Zfp296

Supplementary Table 4
Oligonucleotide sequences used

shRNA target sequences

HDAC5	CATCGCTGAGAACGGCTTTAC
GNAS	TCGGGATGAGTTTCTGAGAAT
LacZ	CCAACGTGACCTATCCCATTA
SIK2	CTTGTTGGTGGAACGTCTAAA
SIK3	CGCACGGAAGTTATGGAAGAT
MEF2C	CCCTATGAATCTAGGAATGAA
CRTC1	ATAGGTCACCTGTCCGATAAT
CRTC2	CAAGGTGTAGAGGGAAATCTT
CRTC3	GACAATGTAGCACTGAATTAA

sgRNA target sequences

HDAC4 #1	TGACGTGTAGAGAGGAAGTG
HDAC4 #2	ACTTACCCATACCAGTAGCG
GNAS #1	CCTCGGCAACAGTAAGACCG
GNAS #2	GATCCTCATCTGCTTCACAA

RT-qPCR primer pairs

	**Forward primer**	**Reverse primer**
SOST	GCCTCATCTGCCTACTTGTG	CTGTGGCATCATTCCTGAAG
RANKL	GCTGGGCCAAGATCTCTAAC	GTAGGTACGCTTCCCGATGT
ß-actin	CCTCTATGCCAACACAGTGC	ACATCTGCTGGAAGGTGGAC
CITED1	CCAACCTTGGAGTGAAGGAT	CCAGAGGAGCTAGTGGGAAC
CRTC1	TCTGCAGACCAGGAGAACAC	GTGGATGTTGGTGAGGTCAG
CRTC2	CCATAGTCACCCATCACTGC	GCACTCAGGACAGGAGATGA
CRTC3	ATGGGTTTCTGTGATGGTGA	ACAGGGACTGGATCTCCTTG
FAM69C	TATTAGCCACATTGCCCTCA	ATGGCGAAGTTCTCAGGTTT
ADAMTS1	GAAACCATGCTCGTAGCTGA	AATTCCTAATGCTGGGATGC
WNT4	GGCCTTTGTATACGCCATCT	CACAGCCACACTTCTCCAGT
KLHL30	AGGTGCAATCTCAACACAGC	GTAGGCCTCCATCTCCACAT
DUSP6	CATGCAGAAGCTCAACCTGT	AGGGTCCTTTCGAAGTCAAG
CD200	GAGCTGGGACTCTGGAACTC	GAGGGTAAGGCAAGCTGTTC
VDR	ACACTGCAGACCTACATCCG	AGCCAGCTTCTGGATCATCT
NR4A2	ATCTCCTGACCGGCTCTATG	TGGGTTGGACCTGTATGCTA
NUAK1	GTGGATGCTGATGGTGAATC	TGCCAAGAGTGGAGACTCAG
PDGFA	CGAAGTCAGATCCACAGCAT	GGGCTCTCAGACTTGTCTCC
MEF2C	ATCAGCAGGCAAAGATTGTG	CTGTTATGGCTGGACACTGG

Primer pairs for ChIP qPCR (RANKL nomenclature is per Onal et al, 2015)

	Forward primer	Reverse primer
SOST +45kB	GAGCCTGGTCTCATTTGTTG	CCTCTCTAGGATGGCAGCAT
SOST promoter	CGCTGTGGTATGCTAACTGG	CTTACAAGTCGAGGCAGGTG
MEOX promoter	CCTCTGGGCAATTTGTCTCT	CTCCAGGGATTGAGAGAAGG
RANKL D2	CTTGGAAGGACTCCAGGAAA	CCTTTCTCAGAGCACACTGG
RANKL D3	AAATCCCATTTGCTTTCCAG	GAGCTGTGTCCTAGAAGAATTGTC
RANKL D4	TGGGAGACTCAGTTGTTGCT	TGTTGTTGGTTCGTTGTCCT
RANKL D5	GATGGAGTCAGGATGCACAG	GAGCCCTGAGAACAGTGTGA
RANKL D6	GAAGAGAACATTGCTGGTTGC	TAAGGATGCTTTCCCAGCTC
RANKL D7	CACCTGTAATTCTAGCACGCA	TCACGCTCCTCTCAAATTCA
RANKL T1	TGGTCCAGGTCAAGCAATAA	GGCAACACAAACCTCCTGTA
RANKL T2	CCTCTGGGAGCAAATGAGAG	GGTGCATCTGTGGATGGTAA
RANKL T3	CCTTGAATTCTTTGGACTGGA	TACACTGTCCTTTCCTTGCG

Supplementary Methods
Small molecule SIK inhibitors:

Synthesis of YKL-04-114 and YKL-05-093 is shown below. Readily accessible compound **1** was reacted with 2,6-dimethylaniline to give compound **2**. Coupling of **2** with isocyanate provided compound **3** in good yield. Ring-closing reaction using tetrabutylammonium hydroxide afforded common intermediate **4** in good yield. By varying anilines and isocyanates used in the procedure described above, various analogues of **4** could be synthesized in multi-gram scale, which enabled the generation of a focused library of inhibitors for structure-activity relationship study. Finally, acid-assisted coupling of intermediate **4** with respective aniline tail gave rise to YKL-04-114 and YKL-05-093 in good yields. For *in vitro* studies, compounds were dissolved in DMSO at 10 mM stocks. For *in vivo* studies, YKL-05-093 was dissolved in PBS plus 25 mM HCl, and solvent was used as vehicle control.

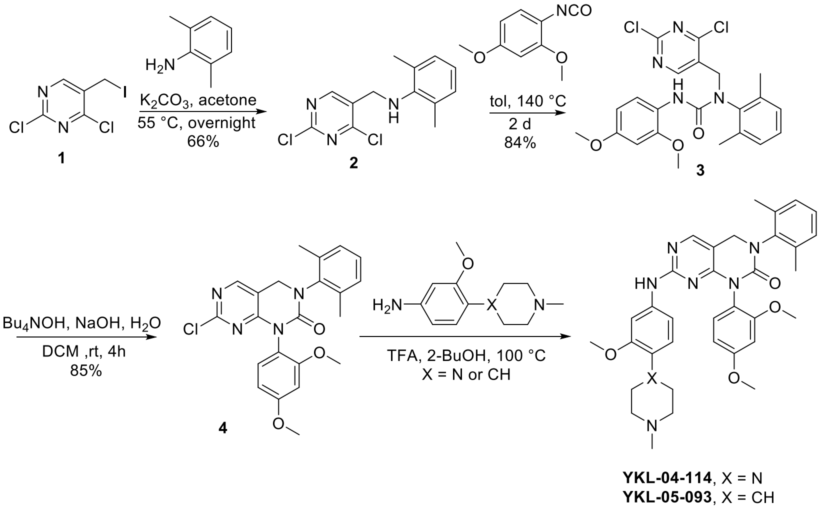

YKL-04-114, X = N
YKL-05-093, X = CH

Unless otherwise noted, reagents and solvents were used as received from commercial suppliers. Proton nuclear magnetic resonance spectra (^{1}H NMR) were obtained on Bruker AVANCE spectrometer at 500 MHz for proton. Carbon nuclear magnetic resonance spectra (^{13}C NMR) were obtained on Bruker AVANCE spectrometer at 125 MHz for carbon. Spectra are given in ppm (δ) and coupling constants, *J*, are reported in Hertz. The solvent peak was used as the reference peak for proton and carbon spectra. High-resolution mass spectra (HRMS) were obtained with an Agilent ESI-TOF mass spectrometer under positive electrospray ionization (ESI^+) conditions at the Small Molecule Mass Spectrometry Facility of Harvard University. NMR data for each compound is shown in Supplemental Figure 9. Starting material 2,4-dichloro-5-(iodomethyl)pyrimidine **1** was obtained according to reported procedures (1, 2).

N-((2,4-dichloropyrimidin-5-yl)methyl)-2,6-dimethylaniline (2)

A mixture of 2,4-dichloro-5-(iodomethyl)pyrimidine (**1**) (7.0 g, 24.2 mmol), 2,6-dimethylaniline (3.8 g, 31.4 mmol), K_2CO_3 (5.0 g, 36.2 mmol) in acetone (60 mL) was stirred at 55 °C overnight. The solvent was removed and the residue was

extracted with EtOAc (150 mL × 3). The combined organic phase was washed with brine (80 mL × 3), dried with Na_2SO_4, filtered, and concentrated. The residue was purified by column chromatography on silica gel (petroleum ether / EtOAc = 8/1, 4/1, 1/1) to get *N*-((2,4-dichloropyrimidin-5- yl)methyl)-2,6-dimethylaniline (**2**) as a light brown solid (4.5 g, yield 66%). ^{1}H NMR (500 MHz, $CDCl_3$) δ 8.55 (s, 1H), 6.95 (d, *J* = 7.4 Hz, 2H), 6.83 (t, *J* = 7.5 Hz, 1H), 4.13 (s, 2H), 3.28 (s, 1H), 2.18 (s, 6H); ^{13}C NMR (125 MHz, $CDCl_3$) δ 161.5, 159.7, 159.3, 144.1, 130.7, 130.4, 129.2, 123.5, 46.6, 18.3; HRMS (ESI$^+$, m/z), calcd. for $C_{13}H_{14}Cl_2N_3$ ([M + H]$^+$) 282.0559, found 282.0557.

1-((2,4-dichloropyrimidin-5-yl)methyl)-3-(2,4-dimethoxyphenyl)-1-(2,6-dimethylphenyl)urea (3)
A round bottomed flask with a Dean-Stark apparatus was charged with *N*-((2,4-dichloropyrimidin-5-yl)methyl) -2,6-dimethylaniline (**2**) (3.0 g, 10.6 mmol), 1-isocyanato-2,4-dimethoxybenzene (2.5 g, 14.0 mmol), toluene (3 mL). The mixture was stirred at 130 °C for 2 d, cooled to room temperature, and concentrated. The residue was purified by column chromatography on silica gel (petroleum ether / EtOAc = 4/1, 2/1, 1/1, EA) to get 1-((2,4-dichloropyrimidin-5-yl)methyl)-3-(2,4-dimethoxyphenyl)-1-(2,6-dimethylphenyl)urea (**3**) as a light brown solid (4.1 g, yield 84%). ^{1}H NMR (500 MHz, $CDCl_3$) δ 8.86 (s, 1H), 7.79 (d, *J* = 8.8 Hz, 1H), 7.17 (dd, *J* = 8.4, 6.7 Hz, 1H), 7.10 (d, *J* = 7.5 Hz, 2H), 6.44 – 6.36 (m, 2H), 6.29 (d, *J* = 2.7 Hz, 1H), 4.84 (s, 2H), 3.69 (s, 3H), 3.51 (s, 3H), 2.06 (s, 6H); ^{13}C NMR (125 MHz, $CDCl_3$) δ 162.7, 161.9, 159.3, 156.3, 154.8, 149.9, 137.9, 137.2, 129.7, 129.2, 121.3, 121.1, 103.9, 98.7, 55.6, 45.9, 18.2; HRMS (ESI$^+$, m/z), calcd. for $C_{22}H_{23}Cl_2N_4O_3$ ([M + H]$^+$) 461.1142, found 461.1134.

7-chloro-1-(2,4-dimethoxyphenyl)-3-(2,6-dimethylphenyl)-3,4-dihydropyrimido[4,5-d]pyrimidin-2(1*H*)-one (4)
To the solution of 1-((2,4-dichloropyrimidin-5-yl)methyl)-3-(2,4-dimethoxyphenyl)-1-(2,6-dimethylphenyl)
urea (**3**) (3.1 g, 6.7 mmol) in DCM (20 mL) was added Bu_4NOH (174 mg, 0.67 mmol), NaOH (474 mg, in 2 mL H_2O, 11.8 mmol). The mixture was stirred at room temperature for 4 h. The final mixture was diluted with H_2O (20 mL), and extracted with DCM (80 mL × 3). The combined organic phase was washed with brine (50 mL × 2), dried with Na_2SO_4, filtered, and concentrated. The residue was purified by column chromatography on silica gel (eluting with DCM/MeOH = 20/1) to give 7-chloro-1-(2,4-dimethoxyphenyl)- 3-(2,6-dimethylphenyl)- 3,4-dihydropyrimido[4,5-d]pyrimidin-2(1*H*)-one (**4**) as an off-white solid (2.4 g, yield 85%). ^{1}H NMR (500 MHz, $CDCl_3$) δ 8.07 (d, *J* = 1.1 Hz, 1H), 7.15 – 6.98 (m, 4H), 6.49 (d, *J* = 7.9 Hz, 2H), 4.63 (d, *J* = 15.1 Hz, 1H), 4.57 (d, *J* = 15.1 Hz, 1H), 3.76 (s, 3H), 3.67 (s, 3H), 2.23 (s, 3H), 2.22 (s, 3H); ^{13}C NMR (125 MHz, $CDCl_3$) δ 161.0, 160.2, 159.6, 156.1, 153.8, 150.5, 138.6, 135.8, 130.9, 129.0, 128.9, 128.3, 117.1, 109.8, 104.5, 99.7, 55.8, 55.5, 45.9, 17.8, 17.6; HRMS (ESI$^+$, m/z), calcd. for $C_{22}H_{22}ClN_4O_3$ ([M + H]$^+$) 425.1375, found 425.1370.

YKL-04-114[1]

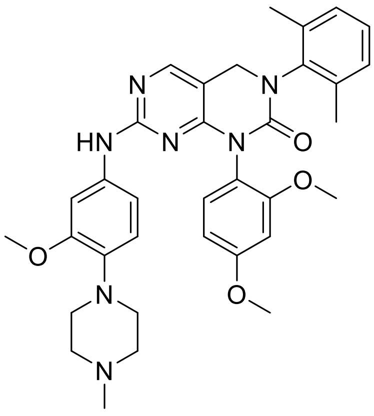

A mixture of 7-chloro-1-(2,4-dimethoxyphenyl)- 3-(2,6-dimethylphenyl)- 3,4-dihydropyrimido[4,5- d]pyrimidin-2(1*H*)-one (**4**) (10 mg, 0.024 mmol), 3-methoxy-4-(4-methylpiperazin-1-yl)aniline (7.8 mg, 0.035 mmol), and TFA (5.5 mg, 0.048 mmol) in 2-BuOH (0.5 mL) was stirred at 100 °C overnight. The reaction was cooled and concentrated. The residue was purified by prep-HPLC (MeOH/H_2O 5:95 – 100:0), followed by column chromatography on silica gel (0-10% MeOH in DCM) to afford **YKL-04-114** as a white solid (8.0 mg, 56%). ^{1}H NMR (500 MHz, DMSO-d_6) δ 9.17 (s, 1H), 8.15 (s, 1H), 7.30 – 7.09 (m, 4H), 6.98 (d, *J* = 8.7 Hz, 1H), 6.92 (s, 1H), 6.72 (d, *J* = 2.7 Hz, 1H), 6.63 (dd, *J* = 8.6, 2.7 Hz, 1H), 6.45 (d, *J* = 8.8 Hz, 1H), 4.67 (d, *J* = 14.5 Hz, 1H), 4.54 (d, *J* = 14.5 Hz, 1H), 3.86 (s, 3H), 3.67 (s, 3H), 3.63 (s, 3H), 2.98 – 2.79 (m, 4H), 2.65 – 2.51 (m, 4H), 2.29 (s, 3H), 2.26 (s, 3H), 2.23 (s, 3H); ^{13}C NMR (125 MHz, DMSO-d_6) δ 160.7, 159.2, 157.9, 157.0, 154.4, 152.2, 151.2, 140.1, 136.3, 136.2, 135.5, 132.0, 128.9, 128.1, 119.2, 117.8, 111.1, 105.2, 104.3, 101.4, 99.8, 56.3, 55.9, 55.7, 55.2, 50.4, 46.0, 17.8, 17.6; HRMS (ESI$^+$, m/z), calcd. for $C_{34}H_{40}N_7O_4$ ([M + H]$^+$) 610.3136, found 610.3124.

YKL-05-093

[1] For compounds YKL-05-093 and YKL-04-114, it is possible that atropisomers exist due to the restricted rotation of the C-N bond between the dihydropyrimidinone core and the dimethylphenyl substituent. Crystallization of both compounds was not successful. Chiral HPLC was also attempted using several conditions, but no sign of atropisomers were observed. This suggests that even if atropisomers exist in these molecules, the rotation energy barrier of this C-N bond could possibly be lower than 20 kcal/mol or fall into the region between 20-30 kcal/mol, which would make the separation of these atropisomers nearly impossible (reference: Revealing Atropisomer Axial Chirality in Drug Discovery, Laplante, Steven R.; Edwards, Paul J.; Fader, Lee D.; Jakalian, Araz; Hucke, Oliver; *ChemMedChem*, **2011**, Vol.6(3), pp.505-513). As a result, these compounds were developed as either a single compound or as an unseparated mixture for SIK inhibition.

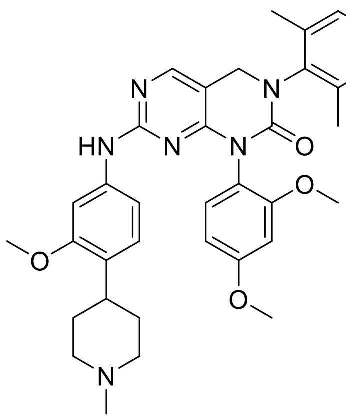

A mixture of 7-chloro-1-(2,4-dimethoxyphenyl)- 3-(2,6-dimethylphenyl)- 3,4-dihydropyrimido[4,5- d]pyrimidin-2(1*H*)-one (**4**) (100 mg, 0.24 mmol), 3-methoxy-4-(1-methylpiperidin-4-yl)aniline (78 mg, 0.35 mmol), and TFA (55 mg, 0.48 mmol) in 2-BuOH (5 mL) was stirred at 100 °C overnight. The reaction was cooled and concentrated. The residue was purified by prep-HPLC (MeOH/H_2O 5:95 – 100:0), followed by column chromatography on silica gel (0-10% MeOH in DCM) to afford **YKL-05-093** as a white solid (127 mg, 89%). ^{1}H NMR (500 MHz, DMSO-d_6) δ 9.17 (s, 1H), 8.09 (s, 1H), 7.16 – 7.05 (m, 4H), 6.95 (d, J = 8.4 Hz, 1H), 6.85 (s, 1H), 6.68 – 6.59 (m, 2H), 6.55 (dd, J = 8.6, 2.6 Hz, 1H), 4.60 (d, J = 15.4 Hz, 1H), 4.47 (d, J = 14.7 Hz, 1H), 3.78 (s, 3H), 3.60 (s, 3H), 3.54 (s, 3H), 2.83 – 2.72 (m, 2H), 2.60 (tt, J = 11.2, 3.7 Hz, 1H), 2.19 (s, 3H), 2.16 (s, 3H), 2.12 (s, 3H), 1.87 (td, J = 11.5, 2.7 Hz, 2H), 1.57 – 1.40 (m, 4H); ^{13}C NMR (125 MHz, DMSO-d_6) δ 160.8, 159.3, 158.0, 157.0, 156.7, 154.4, 151.2, 140.1, 139.8, 136.3, 136.2, 132.0, 129.0, 128.1, 127.0, 126.0, 119.1, 111.0, 105.2, 102.3, 101.7, 99.8, 56.6, 56.3, 55.9, 55.6, 46.7, 46.0, 34.2, 32.3, 17.8, 17.6; HRMS (ESI$^+$, m/z), calcd. for $C_{35}H_{41}N_6O_4$ ([M + H]$^+$) 609.3184, found 609.3176.

REFERENCES

1. Brossmer R, Rohm E. [Preparation and properties of 2,6-dichloro-5-chloromethylpyrimidine]. Justus Liebigs Annalen der Chemie. 1966 Mar;692:119-33. PubMed PMID: 5974228. Darstellung und Eigenschaften von 2.6-Dichlor-5-chlormethylpyrimidin.

2. Ren PZ, G.; You, S.; Sim, T.; Gray, N. S.; Xie, Y.; Wang, X.; He, Y. Compositions and methods for FGF receptor kinases inhibitors. PCT Int Appl. 2007 29 Nov 2007;2007/136465 A2.
